# Biallelic variants in *DNAJC7* cause familial amyotrophic lateral sclerosis with the TDP-43 pathology

**DOI:** 10.1007/s00401-025-02899-y

**Published:** 2025-08-13

**Authors:** Toru Yamashita, Osamu Yokota, Daiki Ousaka, Hongming Sun, Takashi Haraguchi, Ricardo Satoshi Ota-Elliott, Chika Matsuoka, Tomohito Kawano, Hanae Nakashima-Yasuda, Yusuke Fukui, Yumiko Nakano, Ryuta Morihara, Masato Hasegawa, Yasuyuki Hosono, Seishi Terada, Manabu Takaki, Hiroyuki Ishiura

**Affiliations:** 1 Department of Neurology, Okayama University Graduate School of Medicine, Dentistry and Pharmaceutical Sciences, 2-5-1 Shikatacho, Okayama, 700-8558 Japan; 2 Department of Neuropsychiatry, Okayama University Graduate School of Medicine, Dentistry and Pharmaceutical Sciences, 2-5-1 Shikatacho, Okayama, 700-8558 Japan; 3 Okayama University Medical School, Okayama, Japan; 4 Department of Psychiatry, Kinoko Espoir Hospital, Okayama, Japan; 5 Department of Pharmacology, Okayama University Graduate School of Medicine, Dentistry and Pharmaceutical Sciences, Okayama, 700-8558 Japan; 6 Department of Neurology, National Hospital Organisation Minami-Okayama Medical Centre, Okayama, 701-0304 Japan; 7 Department of Psychiatry, Zikei Hospital, Okayama, 702-8508 Japan; 8 Department of Brain and Neurosciences, Tokyo Metropolitan Institute of Medical Science, Setagaya, Tokyo 156-8506 Japan

**Keywords:** Amyotrophic lateral sclerosis, Heat shock protein, *DNAJC7*, TDP-43, Live-cell imaging, Zebrafish disease model

## Abstract

Amyotrophic lateral sclerosis (ALS) is a fatal neurodegenerative disorder characterized by the progressive degeneration of motor neurons. ALS pathology primarily involves the failure of protein quality control mechanisms, leading to the accumulation of misfolded proteins, particularly TAR DNA-binding protein 43 (TDP-43). TDP-43 aggregation is a central pathological feature of ALS. Maintaining protein homeostasis is critical and facilitated by heat shock proteins (HSPs), particularly the HSP40 family, which includes co-chaperones such as DNAJC7. Here, we report a family with three siblings affected by ALS who carry a homozygous c.518dupC frameshift variant in *DNAJC7*, a member of the HSP40 family. All three patients exhibited progressive muscle weakness, limb atrophy, bulbar palsy, and respiratory failure. Pathological examination revealed degeneration of both upper and lower motor neurons, with phosphorylated TDP-43-positive neuronal cytoplasmic inclusions in the frontal and temporal cortices. Immunoblot analysis were consistent with a type B pattern of phosphorylated TDP-43 in the precentral gyrus. Immunohistochemistry and RNA sequencing analyses demonstrated a substantial reduction in *DNAJC7* expression at both the protein and RNA levels in affected brain regions. In a TDP-43 cell model, *DNAJC7* knockdown impaired the disassembly of TDP-43 following arsenite-induced stress, whereas *DNAJC7* overexpression suppressed the assembly and promoted the disassembly of arsenite-induced TDP-43 condensates. Furthermore, in a zebrafish ALS model, *dnajc7* knockdown resulted in increased TDP-43 aggregation in motor neurons and reduced survival. To the best of our knowledge, this study provides the first evidence linking biallelic loss-of-function variants in *DNAJC7* to familial ALS with TDP-43 pathology.

**Supplementary Information:**

The online version contains supplementary material available at 10.1007/s00401-025-02899-y.

## Introduction

Amyotrophic lateral sclerosis (ALS) is a devastating neurodegenerative disorder characterized by the progressive degeneration of motor neurons. It typically manifests in middle age or later, with the selective loss of motor neurons leading to muscle weakness, limb atrophy, dysarthria, and dysphagia. Ultimately, ALS causes respiratory failure, resulting in death within 5 years of symptom onset [10]. Approximately 10% of ALS cases are inherited, with known genetic mutations, including those in *Cu/Zn superoxide dismutase (SOD1)* [1, 42], *TAR DNA-binding protein 43 kDa* (*TARDBP*) encoding TDP-43 [2, 29, 44], *fused in sarcoma* (*FUS*) [34, 48], and the hexanucleotide repeat expansion in the *C9orf72* gene [16, 41]. Notably, mislocalised, cytoplasmic accumulations of hyperphosphorylated, insoluble TDP-43 aggregates are found in selected neurons and glial cells in nearly all sporadic ALS [37] cases. In the remaining familial and sporadic cases without TDP-43 pathology, misfolded SOD1 inclusions or FUS-positive inclusions have been reported in motor neurons [17, 19, 26]. In motor neurons, which rarely undergo cell division and possess long axons, the accumulation of abnormal proteins is strongly implicated in cellular aging and cytotoxicity. Consequently, the failure of protein quality control, resulting in the misfolding of proteins such as TDP-43, FUS, and SOD1, is now considered a common pathological feature of ALS.

In this study, we analysed a consanguineous family with multiple individuals affected by ALS using a linkage-based approach. Linkage and sequence analyses identified a homozygous frameshift variant in *DNAJC7*, a gene not previously implicated in autosomal recessive familial ALS (FALS). Neuropathological examination revealed degeneration of both upper and lower motor neurons with phosphorylated TDP-43-positive inclusions, and a type B pattern of phosphorylated TDP-43 was confirmed by immunoblotting. Transcriptomic and immunohistochemistry analyses using autopsied brains further showed a marked reduction of *DNAJC7* expression at both the RNA and protein levels. Knockdown studies in cultured cells and a zebrafish model supported impaired regulation of TDP-43 by the loss of function of *DNAJC7*.

*DNAJC7* encodes a co-chaperone belonging to the HSP40 (heat shock protein 40 kDa) family. HSPs are central to the protein quality control machinery and are upregulated in response to various stressors such as heat, oxidative stress, and inflammation. Among them, proteins belonging to the HSP70 and HSP90 families assist protein folding in an ATP-dependent manner, while HSP40 proteins modulate the activity of HSP70 by stimulating the ATP hydrolysis activity of HSP70 [8, 30]. Of note, a large-scale exome-wide association analysis has demonstrated heterozygous loss-of-function variants in *DNAJC7* as a genetic risk factor for ALS [18]. Our current findings suggest that biallelic variants in *DNAJC7* cause autosomal recessive FALS with TDP-43 pathology, likely through disrupted TDP-43 homeostasis, thereby reinforcing its role in motor neuron disease pathogenesis.

## Materials and methods

### Human brains

We analysed brain tissue samples from fourteen autopsied individuals, including two patients with FALS (IV-1 and IV-4), three patients with sporadic ALS (SALS), three patients with frontotemporal lobar degeneration (FTLD)-TDP, and six neuropathologically normal individuals without any neurological diseases. Autopsies were conducted after written informed consent was obtained from the family members of the deceased. Frozen precentral gyrus tissue was used for RNA-seq and biochemical analyses. These tissue samples were snap-frozen in liquid nitrogen and stored at -80 °C until further analysis. The study was conducted in accordance with the Ethical Guidelines for Human Genome/Gene Analysis Research and the Guidelines for Medical and Health Research Involving Human Subjects, as set forth by the Japanese government. The use of human biological samples was approved by the Ethics Review committees of Okayama University, the National Hospital Organisation Minami-Okayama Medical Centre, Zikei Hospital, and the Tokyo Metropolitan Institute of Medical Science.

### Short-read sequencing and Sanger sequencing

For DNA analysis, written informed consent was obtained from patient IV-5 and the spouses of deceased patients IV-1 and IV-4, as well as from V-6, the offspring of IV-5, with approval from the Research Ethics committee of Okayama University (#2109-031, #2106-018). Genomic DNA was extracted from peripheral blood leucocytes (IV-1, IV-5, and V-6) or autopsied brain tissue (IV-4). Exome sequencing for the three patients was conducted using the HiSeq X Ten platform (Illumina) with the SureSelect V6 kit (Agilent). Short reads were aligned to the hg38 reference genome using bwa-mem2 v2.2.1 [49]. The aligned reads were processed, and variant calling was performed in accordance with the GATK variant calling best practices (v4.3.0). Annotations were added using ANNOVAR [50]. Rare variants were defined as those with minor allele frequencies (MAF) of < 0.005 across all populations of the gnomAD database. Additionally, short-read whole-genome sequencing was performed for patient IV-5, as described above. Structural variants were detected using Manta v1.6.0 [15]. Variants are described in accordance with the recommendations of the HGVS sequence variant nomenclature. Variants in *DNAJC7* were confirmed by direct nucleotide sequencing with the following primers: 5′-TATCCTGTGGTGAAGCTGCC-3′ and 5′-TGGACCCAAAGACCTCATTGAG-3′.

### Linkage analysis

Using 50 in-house exome and whole-genome sequencing datasets, we selected 11,028 biallelic single nucleotide variants (SNVs) with a call rate greater than 0.9 and allele frequencies ranging from 0.1 to 0.9. If multiple variants were located within a 0.1 cM bin, only one variant was retained for analysis. After extracting the SNV data from the three patients, parametric linkage analysis was performed using Allegro version 2 [23] under an autosomal recessive model with complete penetrance. The disease allele frequency was set at 0.001.

### RNA-seq

RNA samples from two FALS cases (IV-1 and IV-4) and four neuropathologically normal control individuals were analysed. Ribosomal RNA (rRNA) was depleted from total RNA using the Ribo-Zero Plus kit, and cDNA was reverse transcribed using random primers, as described in the Illumina Stranded Total RNA Prep kit protocol. Short reads generated by the NovaSeq 6000 platform were preprocessed using FastQC (v0.11.7) and Trimmomatic (v0.38) [7]. The reads were then aligned to the hg38 reference genome using HISAT2 (v2.1.0) [31] and Bowtie2 (v2.3.4.1) [35]. Transcripts were assembled, and expression levels were estimated with StringTie (v2.1.3b) [40]. Differential expression analysis was conducted using comparison pairs of FALS versus control samples. Statistical analysis was performed using fold change and the exactTest method implemented in the edgeR package for each comparison pair. Significant differentially expressed genes were defined as those with an absolute fold change (|fold change|) ≥ 2 and an exactTest raw *p* value < 0.05.

### Conventional neuropathological examination

Brain tissue samples from two patients with FALS (IV-1 and IV-4) and six neuropathologically normal individuals were fixed postmortem in 10% formaldehyde and embedded in paraffin. Sections (10 μm thickness) from various regions, including the frontal, temporal, parietal, occipital, insular, and cingulate cortices; hippocampus; amygdala; basal ganglia; midbrain; pons; medulla oblongata; cerebellum; and spinal cord, were prepared. These sections were then stained with H&E, Klüver–Barrera, Gallyas–Braak silver, and modified Bielschowsky silver stains.

### Immunohistochemistry

Paraffin-embedded sections were immunostained using the immunoperoxidase method with 3, 3′-diaminobenzidine tetrahydrochloride as the chromogen. The following primary antibodies were used: DNAJC7 (rabbit monoclonal, 1:200, Abcam, Cambridge, UK), tau phosphorylated at Ser-202 (AT8, mouse monoclonal, 1:1000, Innogenetics, Ghent, Belgium), three-repeat (3R) tau (RD3, mouse monoclonal, 1:2000, Millipore, Temecula, CA, USA), four-repeat (4R) tau (anti-4R tau, rabbit polyclonal, 1:2000, Cosmo Bio Co., Tokyo, Japan), phosphorylated TDP-43 (pS409/410-2, rabbit polyclonal, 1:5000, Cosmo Bio), FUS (HPA008784, rabbit polyclonal, 1:200, Sigma-Aldrich, St. Louis, MO, USA), phosphorylated neurofilament (SMI31, mouse monoclonal, 1:1000, Sternberger, Lutherville, MD, USA), N-terminus of p62 protein (p62-N, pig polyclonal, 1:100, Progen, Heidelberg, Germany), C-terminus of p62 protein (p62-C, guinea pig polyclonal, 1:500, Progen Biotechnik GmbH), Aβ (11–28) (12B2, mouse monoclonal, 1:100, IBL, Fujioka, Japan), phosphorylated α-synuclein (pSyn#64, mouse monoclonal, 1:5000, Wako Co. Ltd., Osaka, Japan), and annexin A11 (1A3C4, mouse monoclonal, 1:8000, Proteintech, Rosemont, IL, USA). Deparaffinized sections were incubated with 1% H_2_O_2_ in methanol for 20 min to eliminate endogenous peroxidase activity. The sections were then treated with 0.2% TritonX-100 for 5 min and washed in phosphate buffered saline (PBS, pH 7.4). After blocking with 10% normal serum, sections were incubated overnight at 4 °C with the primary antibodies diluted in 0.05 M Tris-HCl buffer (pH 7.2) containing 0.1% Tween and 15 mM NaN3. Following three 10-min washes in PBS, sections were incubated for 1 h with biotinylated anti-rabbit, anti-mouse, or anti-pig secondary antibodies and then with avidin-biotinylated horseradish peroxidase complex (ABC Elite kit, Vector) for 1 h. Peroxidase labeling was visualized using diaminobenzidine-nickel as the chromogen.

### Semiquantitative assessment of neuronal loss and TDP-43-positive pathologies

Neuronal loss associated with gliosis in the cerebral cortex was assessed on H&E- and Klüver–Barrera-stained sections using the grading system established previously [54, 55]. The grading scale was as follows: -, no histopathological alteration; +, slight neuronal loss and gliosis observed only in the superficial layers; ++, obvious neuronal loss and gliosis in cortical layers II and III, often accompanied by spongiosis, with relative preservation of neurons in layers V and VI; +++, pronounced neuronal loss with gliosis in all cortical layers, along with prominent fibrillary gliosis in the adjacent subcortical white matter. The degree of neuronal loss and gliosis in the basal ganglia and brainstem nuclei was similarly assessed [54, 55] according to the following scale: -, no histopathological alteration; ±, mild gliosis, with reduced number of neurons; + mild gliosis and neuronal loss; ++, moderate neuronal loss and gliosis, with no tissue rarefaction; +++, severe neuronal loss, gliosis, and tissue rarefaction. The severity of degeneration in the frontopontine tract, corticospinal tract, dorsal spinocerebellar tract, and posterior funiculus was assessed as follows [54, 55]: −, neither loss of myelin nor glial proliferation; slight myelin loss and gliosis without atrophy of the tract; ++, evident myelin loss and gliosis with slight atrophy of the tract; and +++, evident myelin loss, gliosis, and severe atrophy of the tract.

TDP-43-positive NCIs, glial inclusions, and threads were semiquantitatively assessed on sections stained with pS409/410-2 using the following grading system: −, no lesion; + , one or more lesions in the anatomical region but fewer than one lesion per × 200 visual field; ++, one lesion per × 200 visual field; +++, two to 10 lesions per × 200 visual field; ++++, 11 to 20 lesions per × 200 visual field; +++++, 21 or more lesions per × 200 visual field.

### Immunofluorescent histochemistry for the human brain

Immunofluorescence was performed to assess the co-localisation of *DNAJC7* and TDP-43 in brain tissue from a patient with SALS. Lipofuscin autofluorescence was quenched using TrueBlack (Biotium) to prevent background interference. Sections were incubated overnight at 4 °C with the following primary antibodies: rabbit anti-*DNAJC7* monoclonal antibody (1:50, abcam) and mouse anti-TDP-43 monoclonal antibody (H-8, 1:50, Santa Cruz). The signals were detected using secondary antibodies conjugated with Alexa Fluor (Invitrogen, Carlsbad, CA).

### Western blotting for DNAJC7, HSPA6, HSPA8, and β-actin.

Brain samples (0.5 g) from two patients with FALS (IV-1 and IV-4) carrying the *DNAJC7* variant, three patients with sporadic ALS, and four disease controls were homogenized in T-PER lysis buffer (Thermo Fisher Scientific) using a homogenizer. The homogenates were centrifuged at 15,000 rpm for 30 min at 4 °C, and the resulting supernatants were mixed with Laemmli buffer and heated at 95 °C. Protein samples were separated by sodium dodecyl sulfate–polyacrylamide gel electrophoresis (SDS-PAGE) and transferred to polyvinylidene difluoride (PVDF) membranes (IPVH00010, Merck Millipore, Darmstadt, Germany). Membranes were blocked with 5% skimmed milk in Tris-buffered saline with Tween-20 (TBST) for 1 h at room temperature and incubated overnight at 4 °C with the following primary antibodies: rabbit anti-DNAJC7 (ab179830, 1:5000, Abcam), mouse anti-HSPA6 (68257-1, 1:10,000, Proteintech), rabbit anti-HSPA8 (8444 T, 1:1000, Cell Signaling Technology), and mouse anti-β-actin (A3854, 1:10,000, Sigma-Aldrich). After washing with TBST, membranes were incubated for 1 h at room temperature with horseradish peroxidase (HRP)-conjugated secondary antibodies: goat anti-mouse IgG (SA00001-1, 1:15,000) and goat anti-rabbit IgG (SA00001-2, 1:15,000, both from Proteintech). Signals were developed using Pierce™ ECL Western blotting substrate (Thermo Fisher Scientific) and visualized with ImageQuant LAS 500 (GE Healthcare, Chicago, IL, USA).

### Western blotting for sarkosyl-insoluble phosphorylated TDP-43

Brain samples (0.5 g) from two patients with FALS (IV-1 and IV-4) carrying the *DNAJC7* variant and three patients with FTLD-TDP were homogenized in 20 mL of homogenization buffer (HB: 10 mM Tris-HCl, pH 7.5, 0.8 M NaCl, 1 mM EGTA, and 10% sucrose). Sarkosyl was added to the homogenates (final concentration: 2%), followed by incubation for 30 min at 37 °C. The homogenates were then centrifuged at 27,000*g* for 10 min at 25 °C. The supernatant was further centrifuged at 166,000*g* for 20 min at 25 °C. The resulting pellets were resuspended in 1 mL of extraction buffer containing 1% Sarkosyl by sonication, incubated for 20 min at 37 °C, and centrifuged at 166,500*g* for 5 min at 25 °C. The pellets were then resuspended in 20 mM Tris-HCl pH 7.4 and 150 mM NaCl by sonication. Immunoblotting was performed using an anti-phosphorylated TDP-43 antibody, as previously described [3, 24].

### Plasmid, cell culture, and stable cell line constructions

The vectors were constructed by VectorBuilder Inc. (Supplementary Table 1). The TDP-43 mutant used in this study, referred to as TDP-43ΔNLS, carries point mutations in the nuclear localisation signal (NLS), in which the lysine at position 82, the arginine at position 83, and the lysine at position 84 are all substituted with alanine (K82A, R83A, and K84A). The sequence of this NLS mutant is adopted from previous report [21]. The cell lines used in this study included HEK293T (American Type Culture Collection [ATCC], CRL-11268) and U2OS (ATCC, HTB-96). Routine maintenance of these cell lines followed the guidelines provided by ATCC. Briefly, U2OS and HEK293T cells were cultured in a complete DMEM medium supplemented with 10% fetal bovine serum (FBS) at 37 °C under 5% CO_2_. Lentiviral vectors were produced in HEK293T cells by transfecting pTRE3G-TDP-43∆NLS-Clover and the packaging plasmids pMD2.G and psPAX2 using FuGENE® HD Transfection Reagent (Promega, E2311). After 3 days of transfection, the culture medium containing lentivirus was filtered through a 0.45-μm filter and used to infect U2OS cells. Following 2 days of infection, the medium was replaced with a medium containing puromycin (2 μg/μL) for selection. Stable clones were selected by limited dilution of suspension cells according to the manufacturer's instructions (Lenti-X lentiviral expression system user manual, Takara Bio, Mountain View, CA, USA).

### EGFP co-transfection and fluorescence imaging

To assess transfection efficiency, a pCMV-EGFP-IRES-Puro expression vector was co-transfected with the DNAJC7 constructs. EGFP fluorescence was observed 48 h after transfection. All images were acquired under identical exposure conditions.

### Reverse transcription PCR for HEK293T cells

Total RNA was extracted from transfected HEK293T cells using the RNA extraction kit (PureLink™ RNA Mini Kit, Thermo Fisher Scientific). cDNA was synthesized from 500 ng of total RNA using the cDNA synthesis kit (ReverTra Ace® qPCR RT Master Mix with gDNA Remover, TOYOBO, FSQ-301). Semi-quantitative reverse transcription PCR was performed using primers specific for *Myc*-*DNAJC7* and *GAPDH*. PCR was conducted under three different cycle conditions (10, 20, and 30 cycles) to evaluate the linear range of amplification, and the 20-cycle condition was selected for subsequent data analysis. The primers used were as follows: forward primer, 5′-ATGGAACAAAAACTCATCTC-3′ and reverse primer, 5′-ATGGAACAAAAACTCATCTC-3′ for *Myc-DNAJC7*; forward primer, 5′-TGCACCACCAACTGCTTAGC-3′ and reverse primer, 5′-GGCATGGACTGTGGTCATGAG-3′ for *GAPDH*.

### siRNA transfection

ON-TARGETplus SMARTpool siRNAs (Horizon Discovery) targeting *HSPA1A* (L-005168-00-0005), *HSPB1* (L-005269-00-0005), *BAG2* (L-011961-00-0005), *DNAJC7* (L-019566-01-0005) or the non-targeting pool (D-001810-10-05) were transfected into U2OS cells at a concentration of 3 fmol per 5000 cells using Lipofectamine RNAiMAX transfection reagent (ThermoFisher, 13778075). The transfection was carried out for 3–4 days before experimental analysis.

### Quantitative reverse transcription PCR for siRNA knockdown validation

cDNA was synthesized from total RNA as described above for HEK293T cells. Quantitative reverse transcription PCR was performed using the StepOnePlus Real-Time PCR system (Thermo Fisher Scientific) and TaqMan™ Gene Expression Assays (Applied Biosystems). The following probe sets were used: *HSPA1A* (Hs00359163_s1), *HSPB1* (Hs00356629_g1), *BAG2* (Hs00989341_m1), *DNAJC7* (Hs00268602_m1), and *GAPDH* (Hs02786624_g1) as the endogenous control. All assays were used at a final 1 × concentration according to the manufacturer’s instructions. Gene expression levels were calculated using the ΔΔCt method and normalized to *GAPDH* expression.

### Live-cell imaging

Stable U2OS cells were plated onto a 6-well plate (FALCON, 353046) in DMEM medium without phenol red. The cells were induced by doxycycline (100 ng/mL) to express TDP-43ΔNLS-Clover and incubated for 24 h. For the first hour, U2OS cells were treated with sodium arsenite (250 µM), and nine images per well were captured every hour using the IncuCyteZOOM (Essen BioScience, Ann Arbour, MI, USA) with a × 20 objective under continuous CO_2_ flow.

### The transgenic zebrafish line and blue light stimulation

Zebrafish (Danio rerio) were maintained at 28–29 ℃ under a 14/10 h light/dark cycle in a filtered freshwater recirculation system. They were fed three times daily, as previously described [53]. All breeding were conducted after approximately 90 dpf when fish were sexually mature. Male and female zebrafish, previously separated, were introduced into a breeding tank, and eggs were subsequently collected. Embryos were raised at 28–29 ℃ and staged in hours post-fertilization according to standard procedures. Approval for the zebrafish research was obtained from the Animal Care and Use Committee at Okayama University. Detailed information about the Tg[mnr2b-hs:opTDP-43h]Tg[mnr2b-hs:EGFP-TDP-43z] transgenic zebrafish line is available in a previous publication [5].

Blue light stimulation was administered from 2 to 6 dpf (approximately 96 h) using an LED array system (BRC Bioresearch Centre) as shown in Fig. 6a. The intensity of blue LED was 0.7 mW per cm^2^, and its wavelength peaked at 456 nm. In contrast, light stimulation using the same protocol was performed from 0 to 6 dpf (approximately 144 h), as shown in Fig. 6c.

### Microscopy

Following light stimulation, all zebrafish larvae were euthanized by exposure to cold water and fixed overnight in PBT (PBS, pH 7.4, containing 0.25% Triton X-100) containing 4% paraformaldehyde. After washing with PBS, permeabilization was carried out with acetone for 10 min at − 30 ℃, followed by DAPI staining. Agarose gel (0.7%) was used to embed the samples on glass slides. 3D images were acquired using a laser confocal microscope (LSM780, Carl Zeiss), as shown in Fig. 6a, c. For evaluation of SB development, zebrafish larvae were anesthetized with 0.02% tricaine and subsequently observed under an APX100 microscope (Olympus) (Fig. 6e).

### *Cas9 target site design, *in vitro* sgRNA synthesis and microinjection of zebrafish embryos*

We identified three sgRNA target sites using the CHOPCHOP Targeter (https://chopchop.cbu.uib.no/). The sequences of the three sgRNAs targeting *dnajc7* were as follows: sgRNA1: 5′-[GATGGACTTAACCAGCGACG]-3′, sgRNA2: 5′-[GCTGACCTTGGAACCGACAG]-3′, and sgRNA3: 5′-[GAAGTTTAAAGAGGTCGGCG]-3′. The sgRNAs were synthesized following the protocols outlined previously [20]. In brief, to generate templates for sgRNA transcription, gene-specific oligonucleotides containing the T7 promoter sequence (5'-TAATACGACTCACTATA-3'), the 20-base target sequence (excluding the PAM sequence), and a complementary region were annealed to a constant oligonucleotide that encodes the reverse-complement of the tracrRNA tail (5′-AAAAGCACCGACTCGGTGCCACTTTTTCAAGTTGATAACGGACTAGCCTTATTTTAACTTGCTATTTCTAGCTCTAAAAC-3′). The ssDNA overhangs were filled in using T4 DNA polymerase (NEB), and the resulting sgRNA template was purified using QIAquick columns (Qiagen). The sgRNAs were transcribed using MAXIscript kits (Ambion), treated with DNase, and cleaned up with RNA Clean & Concentrator Kits (Zymo Research). At the one-cell stage, embryos were microinjected with 250 ng/μL of Cas9 protein and 150 ng/μL of each sgRNA using a pneumatic pico-pump (PV-820, World Precision Instrument).

### Reverse transcription PCR for zebrafish

Total RNA was extracted from zebrafish embryos at 1 dpf using the RNA extraction kit (PureLink™ RNA Mini Kit, Thermo Fisher Scientific). cDNA was synthesized from 500 ng of total RNA using the cDNA synthesis kit (QuantiTest Reverse Transcription Kit, Qiagen). Quantitative reverse transcription PCR was performed using the SYBR Green Master Mix (Takara) on a StepOnePlus Real-Time PCR system (Thermo Fisher Scientific). The primers used to amplify *dnajc7* mRNA were as follows: forward primer, 5′-[CAAAGAACAGGGAAACGCATA]-3′ and reverse primer, 5′-[CATCATCAATGTGGCTGCAC]-3′. Expression levels of *dnajc7* mRNA were normalized to the expression of the housekeeping gene *gapdh*, with the following primers: forward primer, 5′-[CCAAGGCTGTAGGCAAAGTAAT]-3′ and reverse primer, 5′-[GGACTGTCAGATCCACAACAGA]-3′.

### T7 Endonuclease I assay

Genomic DNA was extracted from zebrafish embryos at 1 dpf using a homemade lysis buffer. The embryos were homogenized in lysis buffer [10 mM Tris-HCl (pH 8.0), 50 mM EDTA] containing proteinase K (200 μg/mL) and incubated at 55 ℃ for 2 h. The targeted region of *dnajc7* was PCR-amplified using the following primers: forward primer, 5'-[TGCAAATGTCACATCCATGA]-3' and reverse primer, 5′-[TCTCTCGCATACCCCCATAC]-3'. The PCR products were denatured and reannealed to form heteroduplexes, which were then treated with T7 endonuclease I (New England BioLabs) at 37 ℃ for 30 min. The digested products were analysed using 1.5% agarose gel electrophoresis.

### Quantification of TDP-43 aggregation

To assess the phenotypic consequences of *dnajc7* knockdown (KD), we imaged motor neurons expressing EGFP-tagged TDP-43z, as well as aggregated RFP-tagged opTDP-43h, in zebrafish larvae using confocal laser scanning microscopy. Images were captured under identical conditions, ensuring consistent laser intensity and mechanical gain across all samples. Subsequent image analysis was performed using a custom Python script developed with the assistance of GPT-4. The script utilized the Python Imaging Library and NumPy libraries to quantify the number of pixels corresponding to green (EGFP), red (RFP), and yellow (EGFP merged with RFP) fluorescence in the captured images. Specifically, green pixels were identified where the green channel intensity exceeded that of the red and blue channels; red pixels were identified where the red channel intensity exceeded the green and blue channels; and yellow pixels were identified where both red and green channel intensities were high (R > 200, G > 200, B < 100). The results were compiled and analysed using pandas, and the data were saved to an Excel file for further statistical analysis. The Python code used for image analysis in this study is provided in Supplementary Data 1.

### Mortality rate and SB inflation failure

To evaluate the phenotypic consequences of *dnajc7* knockout (KO), two key parameters—survival rates and the incidence of SB inflation failure were assessed. Zebrafish larvae were monitored daily for survival over a 6-day period of fertilization. The total number of deaths was recorded each day, and the cumulative survival rate was calculated based on the total number of larvae that survived the entire 6-day period. For the assessment of SB inflation failure, larvae were examined under a dissecting microscope to determine the inflation status of the SB at 6 dpf. The number of larvae exhibiting SB inflation failure was counted, and the percentage of larvae with SB inflation failure was calculated relative to the total number of larvae observed.

### Statistical analyses

All quantitative data were analysed using GraphPad Prism (version 9.5.1, GraphPad Software Inc., San Diego, CA, SCR_002798) and are expressed as mean ± SD. Statistical significance between the two groups was assessed using a two-tailed unpaired Student’s *t*-test. For cells with or without TDP-43 condensates after siRNA treatment, normality was checked using the Shapiro–Wilk test. Differences were further evaluated using a non-repeated measures analysis of variance followed by a Bonferroni post hoc test. In zebrafish experiments, comparisons of mRNA expression levels and pixel quantification between the sg control and sg dnajc7 groups (Fig. 6b, c, e) were performed using the Mann–Whitney U-test. Survival rates and the incidence of SB inflation failure (Fig. 6d, e) were compared using Fisher's exact test. Statistical significance was considered at a *p* value of < 0.05.

## Results

### Clinical features of three siblings with ALS

**Fig. 1:**
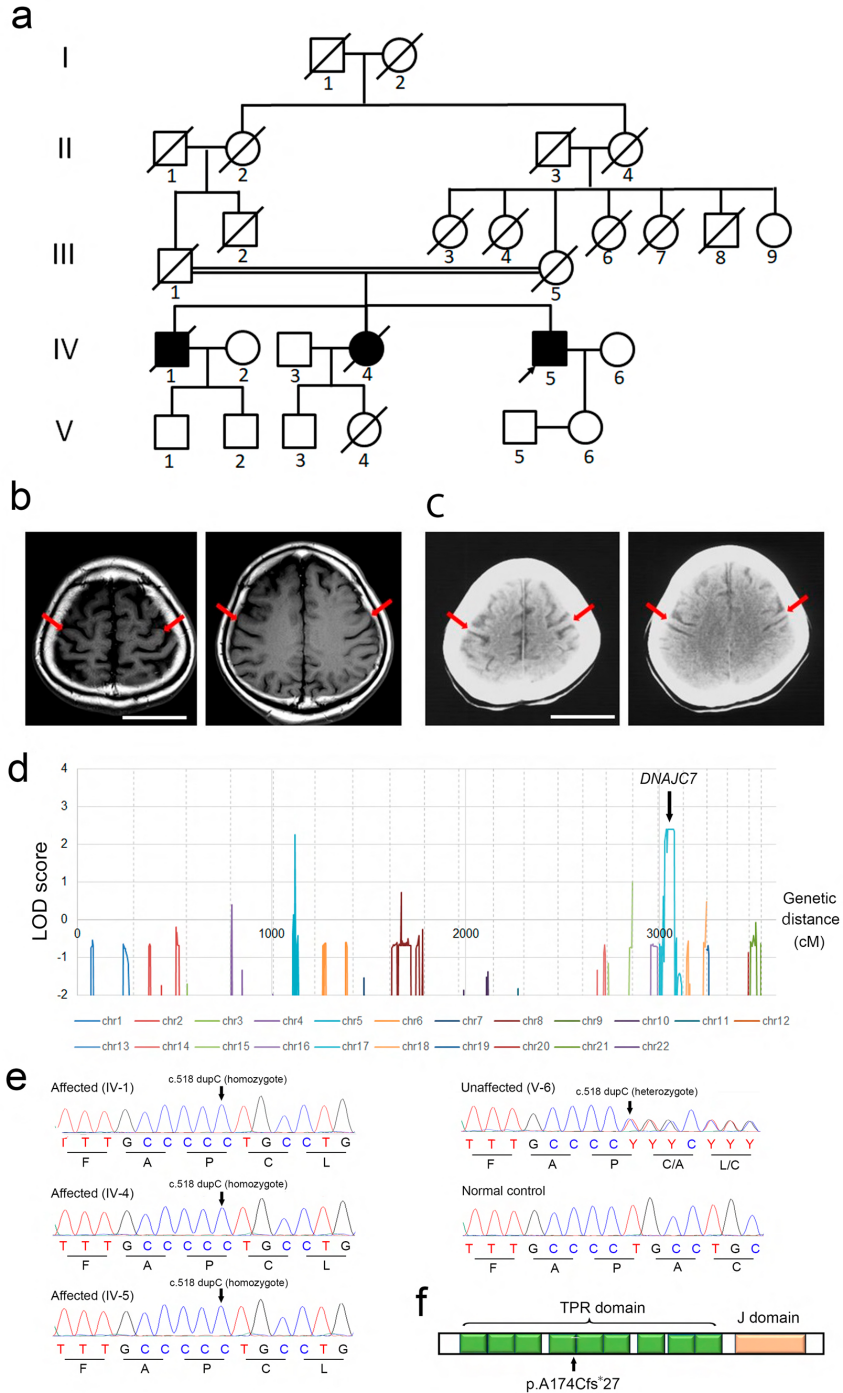
Identification of homozygous *DNAJC7* variants in three patients with ALS. **a** The pedigree chart of a family with three ALS patients. The proband is indicated by an arrow. **b** Brain T1-weighted magnetic resonance images of the proband (IV-5), with red arrows showing characteristic atrophy of the bilateral frontal lobes. Scale bar: 50 mm. **c** Brain computed tomography images of the proband’s older sister (IV-4), with red arrows indicating atrophy of the bilateral frontal lobes. Scale bar: 50 mm. **d** Parametric multipoint LOD scores showing significant peaks on chromosomes 5 and 17, suggesting a genetic linkage. **e** Chromatograms confirming the homozygous c.518dupC variant in *DNAJC7* in all three affected individuals. **f** Schematic representation of the *DNAJC7* protein, with the ALS-associated variant located within the tetratricopeptide repeat domain. ALS, amyotrophic lateral sclerosis; LOD, logarithm of odds

As shown in Fig. 1a, the parents of the three siblings were cousins. Table 1 summarizes the main clinical features of the three siblings. The proband (IV-5) exhibited progressive muscle weakness and atrophy in the upper limbs, beginning at age 65, with neurophysiological examinations confirming a diagnosis of ALS. Brain magnetic resonance imaging revealed diffuse bilateral frontal lobe atrophy (Fig. 1c). At the time of submission, the proband remained alive, dependent on ventilator support due to decreased respiratory function. The elder brother (IV-1) developed progressive muscle weakness and atrophy in the lower limbs, followed by muscle weakness in the upper limbs and bulbar palsy. He passed away 8 years after disease onset, without ventilator support, and underwent autopsy. The elder sister (IV-4), in her early 30 s, presented with muscle weakness in the lower limbs. A brain computed tomography scan revealed diffuse frontal lobe atrophy (Fig. 1b). She passed away at age 40 without ventilator management and was also autopsied.

**Table 1:** Clinical characteristics of the three patients

	Case 1 (IV-1)	Case 2 (IV-4)	Case 3 (IV-5)
Variant	c.518dupC, p.A174Cfs*27	c.518dupC, p.A174Cfs*27	c.518dupC, p.A174Cfs*27
Sex	Male	Female	Male
Age at onset (Y)	46	Early 30 s	65
Age at evaluation (Y)	50	36	66
Age at autopsy (Y)	54	40	−
Initial symptoms	Muscle weakness in lt. LE	Muscle weakness in bil. LE	Muscle weakness in lt. UE
Tongue	Atrophy +; Fasciculation +	Atrophy +; Fasciculation +	Normal
Muscle weakness and muscle atrophy	Generalized	Generalized	UE dominant, distal dominant
Deep tendon reflex	Hyporeflexia in AE	Hyporeflexia in AE	Hyporeflexia in UE, Hyperreflexia in LE
Babinski reflex	+	−	−
Sensory disturbance	None	None	None
Respiratory insufficiency	+	+	None
Cognitive impairment	None	None	None
Brain MRI	n.e.	n.e.	Diffuse frontal lobe atrophy
Needle EMG	n.e.	n.e.	Acute and chronic denervation
Motor NCS	n.e.	n.e.	Decreased amplitude of CMAP
Sensory NCS	n.e.	n.e.	Normal

AE, all extremity; AR, autosomal recessive; CMAP, compound muscle action potential; EMG, electromyography; LE, lower extremity; NCS, nerve conduction studies; n.e., not evaluated; UE, upper extremity; MRI, magnetic resonance imaging; bil., bilateral; lt., left

### Identification of a *DNAJC7* variant in the family

Genomic DNA was extracted from the patients, and linkage analysis using exome data identified a peak with a multipoint logarithm of odds (LOD) score of 2.40 on chromosome 17 (17p12–17q22). This region spanned 44.8 Mb and was delimited by rs12453883 and rs6503820. Additionally, a 10.5 Mb region on chromosome 5, bounded by rs7718898 and rs246105, exhibited a multipoint LOD score of 2.28 (Fig. 1d).

Variants were called from whole-genome sequencing data obtained from IV-5. Rare nonsynonymous variants with allele frequencies < 0.005 in any of the gnomAD subpopulations (v2.1.1 and v.3.1.2) were excluded. No rare variants were found within the candidate region on chromosome 5. In contrast, within the candidate region on chromosome 17, we identified five homozygous rare variants (Supplementary Table 2) after excluding heterozygous calls in *KRTAP1-1*, likely due to the presence of homologous genes. Among these, a homozygous variant in *DNAJC7* (NM_003315.4:c.518dupC, p.Ala174Cysfs*27) was identified, which is not registered in gnomAD (v4.1.0). Variants in *TRPV2*, *HAP1*, *KCNH4*, and *DCAKD* were registered in gnomAD with low MAF, and none of these genes are associated with known diseases. No copy number variants greater than 50 bp were identified in the exonic regions of the candidate regions. The homozygous variant in *DNAJC7* was confirmed in the three affected individuals by Sanger sequencing (Fig. 1e). Given that heterozygous loss-of-function variants in *DNAJC7* have been implicated in ALS [18], we consider the homozygous variant in *DNAJC7* to be causative in this family.

### Pathological analysis of patients with *DNAJC7* variants

**Fig. 2:**
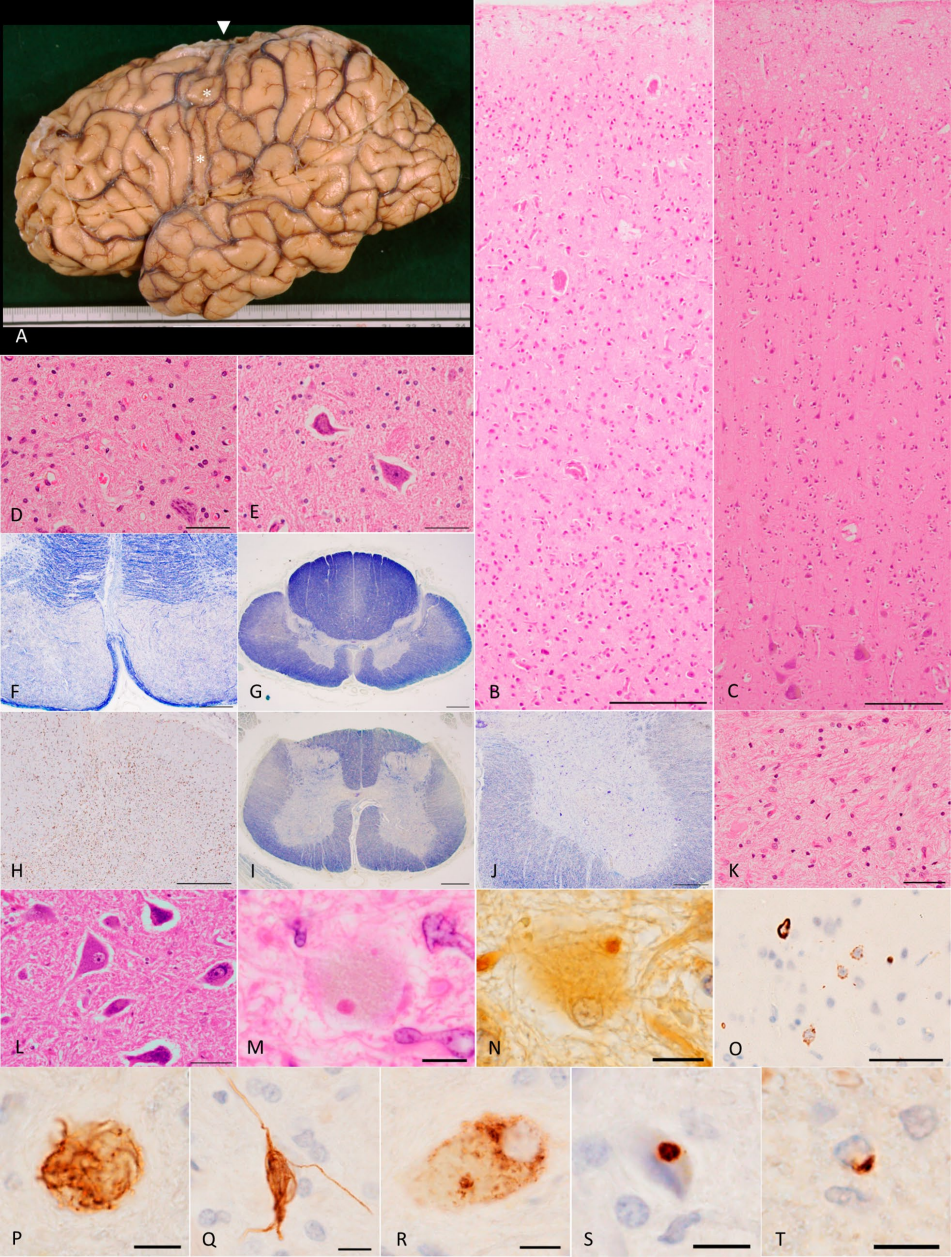
Phosphorylated TDP-43 pathology, Bunina bodies, and severe loss of the upper and lower motor neurons in the older brother (IV-1) with a homozygous c.518dupC frameshift variant in *DNAJC7.* **a** Lateral view of the left hemisphere, showing severe atrophy in the precentral gyrus (asterisks). Arrowhead indicates the top of the precentral gyrus, the width of which is extremely thin. **b** Severe neuronal loss with gliosis in all cortical layers of the primary motor cortex. Betz cells are nearly completely absent. H&E stain. Scale bar: 200 μm. **c** Primary motor cortex in a 68-year-old pathologically normal control case. Betz cells are present in layer V, and the structure of the cerebral cortex is spared. H&E stain. Scale bar: 200 μm. **d** Severe neuronal loss with gliosis in the hypoglossal nucleus. A remaining neuron shows shrinkage of the cell body. H&E stain. Scale bar: 50 μm. **e** Hypoglossal nucleus in a 51-year-old pathologically normal control case. Neither glial proliferation nor shrinkage of neurons is observed. H&E stain. Scale bar: 50 μm. **f** Severe degeneration in the pyramidal tract within the medulla oblongata, as visualized by Klüver–Barrera stain. Scale bar: 500 μm. **g**, **h** Severe loss of myelin (**g**) with microglial activation (**h**) in the lateral tract of the thoracic cord. The dorsal spinocerebellar tract does not exhibit these changes. **g** Klüver–Barrera stain, **h** Iba-1 immunohistochemistry. Scale bars: **g** 1 mm and **h** 500 μm. **i** Degeneration in the lateral tract of the lumbar cord, as shown by Klüver–Barrera stain. Scale bar: 1 mm. **j** Severe loss of anterior horn cells in the lumbar cord, visualized by Klüver–Barrera stain. Scale bar: 500 μm. **k** Severe gliosis with loss of neurons in the anterior horn of the lumbar cord. H&E stain. Scale bar: 50 μm. **l** The Spinal anterior horns in a 67-year-old pathologically normal control case. Neither loss of anterior horn cells nor gliosis is present. H&E stain. Scale bar: 50 μm. **m** Bunina bodies in the lumber anterior horn. H&E stain. Scale bar: 10 μm. **n** Bunina body in the lumbar anterior horn, identified by Cystatin C immunohistochemistry. Scale bar: 10 μm. **o** Phosphorylated TDP-43-positive NCIs in the upper layers of the primary motor cortex. Neurites are barely discernible. pS409/410-2 immunohistochemistry. Scale bar: 50 μm. **p**–**s** Various morphologies of TDP-43-positive NCIs in the lumbar anterior horns. Inclusions include **p** skein-like, **q** neurofibrillary change-like, **r** diffuse granular, and **s** round structures. pS409/410-2 immunohistochemistry. Scale bars: 10 μm. **t** Phosphorylated TDP-43-positive glial cytoplasmic inclusion in the pyramidal tract of the medulla oblongata. pS409/410-2 immunohistochemistry. Scale bar: 10 μm. **c**, **e**, **l** Control cases presented here lacked any neurodegenerative changes except for minimal NFTs with Braak stage I. H&E, hematoxylin–eosin; NCI, neuronal cytoplasmic inclusions; NFTs, neurofibrillary tangles; TDP-43, TAR DNA-binding protein of 43 kDa

Two autopsied cases (IV-1 and IV-4) were examined pathologically. The pathological features of these cases are summarized in Table 2, Fig. 2, and Supplementary Fig. 1. Conventional pathological examination revealed characteristic features of ALS in both cases. Macroscopically, severe atrophy in the precentral gyrus was observed in case IV-1, with corresponding photographic documentation (Fig. 2a). Microscopically, both cases showed severe neuronal loss, gliosis, and tissue rarefaction in all cortical layers of the primary motor cortex (Fig. 2b and Supplementary Fig. 1a). In contrast, the other regions of the frontal and temporal cortices were only minimally affected by neuronal loss. The degeneration of the corticospinal tract in the medulla oblongata and spinal cord was pronounced, with marked myelin loss (Fig. 2f, g, i, Supplementary Figs. 1c, g, h) and glial proliferation (Fig. 2h and Supplementary Fig. 1d). In the hypoglossal nuclei, motor neurons were significantly reduced, and glial proliferation was also observed (Fig. 2d and Supplementary Fig. 1e). Anterior horn cells in the spinal cord were markedly diminished (Fig. 2i, j, and Supplementary Fig. 1i). The remaining neurons appeared shrunken, and gliosis with tissue rarefaction was evident (Fig. 2k and Supplementary Fig. 1i). Some of the surviving neurons contained typical Bunina bodies, which were visible in hematoxylin–eosin (H&E)-stained sections (Fig. 2m and Supplementary Fig. 1k) and were immunolabeled with an anti-cystatin C antibody (Fig. 2n and Supplementary Fig. 1l).

**Table 2:** Distribution of neuronal loss and TDP-43-positive lesions in two autopsy cases

	Case 1 (IV-1)						Case 2 (IV-4)					
Brain weight (g)	1280						1150					
Braak NFT stage	Stage II						Stage I					
Thal Aβ phase	Phase 1						Phase 0					
CERAD neuritic plaque score	0						0					
Lewy body disease	−						−					
Saito AG stage	Stage 0						Stage 0					
Brettschneider ALS-TDP stage	Stage 4						Stage 4					
TDP-43 histologic subtype	Type B						Type B					
FUS pathology	–						–					
Bunina body	Present						Present					
Postmortem interval (h)	n.a						5					
RNA integrity number	8.6						7.4					

		TDP-43 pathology						TDP-43 pathology				
	NL	NCI	NII	LN	SN	GI	NL	NCI	NII	LN	SN	GI
**Cerebral cortex**												
Primary motor cortex	+++	++++	−	−	+	+++	+++	+++++	−	−	+	++
Superior frontal gyrus	−	+++	−	−	+	+	+	+++	−	−	−	++
Middle frontal gyrus	−	+	−	−	−	+	+	+	−	−	−	++
Inferior frontal gyrus	−	+++	−	−	+	+	n.a	+	−	−	−	+
Frontal white matter	−	−	−	−	−	++	−	−	−	−	−	++
Superior temporal gyrus	−	−	−	−	−	−	−	+++	−	−	−	+
Middle temporal gyrus	−	−	−	−	−	−	+	++	−	−	−	−
Inferior temporal gyrus	−	+	−	−	−	−	+	+	−	−	−	−
Lateral occipitotemporal gyrus	−	+	−	−	−	−	−	+++	−	−	−	+
Temporal white matter	−	−	−	−	−	+	−	−	−	−	−	+++
Occipital cortex	−	−	−	−	−	−	n.a	n.a	n.a	n.a	n.a	n.a
Occipital white matter	−	−	−	−	−	−	n.a	n.a	n.a	n.a	n.a	n.a
Cingulate gyrus	−	++++	−	−	−	+	++	+++	−	−	−	+
Insular cortex	−	+++	−	−	−	+++	++	++++	−	−	−	+
**Limbic region**												
Amygdala	−	+++	−	−	−	+	+	+	−	−	−	−
Entorhinal cortex	−	+	−	−	−	−	+	+	−	−	−	−
Subiculum	−	+	−	−	−	−	++	++	−	−	−	++
CA1	−	+	−	−	−	−	+	+	−	−	−	−
CA2	−	−	−	−	−	−	+	−	−	−	−	−
CA3	−	+	−	−	−	−	+	−	−	−	−	−
CA4	−	−	−	−	−	−	+	−	−	−	−	+
Dentate gyrus	−	+	−	−	−	−	−	++	−	−	−	−
**Basal ganglia**												
Caudate nucleus	−	+++++	−	−	−	+	++	+++	−	−	−	+
Putamen	−	+++	−	−	−	−	+++	+++	−	−	−	++
Globus pallidus, internal segment	−	−	−	−	−	++++	+++	+++	−	−	−	+++
Globus pallidus, external segment	−	−	−	−	−	+++	+++	+++	−	−	−	+++
Thalamus	−	+++	−	−	−	+	++	+	−	−	−	++++
Subthalamic nucleus	−	++	−	−	−	+++	−	+++	−	−	−	+++
**Brain stem**												
Oculomotor nucleus	−	−	−	−	−	−	−	−	−	−	−	−
Substantia nigra	−	−	−	−	−	−	−	+	−	−	−	+
Red nucleus	−	−	−	−	−	+	−	+++	−	−	−	+++
Frontopontine tract (cerebral peduncle)	−	−	−	−	−	+	−	−	−	−	−	+++
Corticospinal tract (cerebral peduncle)	+++	−	−	−	−	++	+	−	−	−	−	+++++
Superior cerebellar peduncle	−	−	−	−	−	+	−	−	−	−	−	+
Locus coeruleus	−	−	−	−	+	+	−	−	−	−	−	+
Pontine nucleus	−	−	−	−	−	+	−	−	−	−	−	++
Dorsal vagal nucleus	−	−	−	−	−	−	−	−	−	−	−	−
Hypoglossal nucleus	+++	+++	−	−	−	−	+++	+++	−	−	+	+++
Inferior olivary nucleus	−	+	−	−	−	+	−	++++	−	−	−	+
Corticospinal tract (medulla oblongata)	+++	−	−	−	−	+	+++	−	−	−	−	++
**Cerebellum**												
Molecular cell layer	−	−	−	−	−	−	−	−	−	−	−	−
Purkinje cell layer	−	−	−	−	−	−	−	−	−	−	−	−
Granular cell layer	−	−	−	−	−	−	−	−	−	−	−	−
Dentate nucleus	−	−	−	−	−	−	−	−	−	−	−	−
White matter	−	−	−	−	−	−	−	−	−	−	−	−
**Spinal cord**												
Posterior funiculus	−	−	−	−	−	−	−	−	−	−	−	−
Lateral corticospinal tract	+++	−	−	−	−	+	+++	−	−	−	−	++
Anterior corticospinal tract	+	−	−	−	−	+	++	−	−	−	−	+++
Dorsal spinocerebellar tract	−	−	−	−	−	−	−	−	−	−	−	−
Dorsal nucleus of Clarke	n.a	−	−	−	−	−	n.a	−	−	−	−	+
Anterior horn cells	+++	+++	−	−	+	+	+++	++	+	−	−	+++

NFT, neurofibrillary tangles and threads; CERAD, the Consortium to Establish a Registry for Alzheimer's Disease; AG, argyrophilic grains; −, absent; TDP-43, TAR DNA-binding protein of 43 kDa; FUS, fused in sarcoma; NL, neuronal loss; NCI, neuronal cytoplasmic inclusions; NII, neuronal intranuclear inclusions; LN, long neurites; SN, short neurites; GI, glial inclusions; n.a., not available. The grading system of each lesion was noted in the text

Phosphorylated TDP-43 immunohistochemistry revealed neuronal cytoplasmic inclusions (NCIs) in the frontotemporal cortex, with a particularly prominent accumulation in the primary motor cortex (Fig. 2o and Supplementary Fig. 1m). TDP-43-positive neurites were scarcely observed in the neocortex, leading to the classification of both cases as having TDP-43 type B histology. Notably, no neuronal intranuclear inclusions (NIIs) were detected in the neocortex. Phosphorylated TDP-43-positive NCIs were also scattered in the hypoglossal nuclei (Supplementary Fig. 1n) and the spinal anterior horns (Fig. 2p–s, Supplementary Fig. 1o, p). Additional NCIs were identified in the putamen, subthalamic nucleus, thalamus, red nucleus, and inferior olivary nucleus. A high density of NCIs was observed in the cingulate and insular cortices as well. In case 2 (IV-4), the anterior horn cells in the spinal cord contained a few phosphorylated TDP-43-positive NIIs, which exhibited a fibrous morphology (Supplementary Fig. 1q). Although TDP-43 aggregates were rare in the hippocampus in both cases, they were frequent in the amygdala in case 1 (IV-1) (Supplementary Fig. 1r). Phosphorylated TDP-43-positive glial inclusions were noted in the pyramidal tract, white matter of the frontal and temporal lobes, and several subcortical nuclei (Fig. 2t). These glial lesions often displayed coiled body-like structures (Supplementary Fig. 1s). Both cases were classified as stage 4 according to the Brettschneider staging system for ALS-TDP.

No pathological FUS-positive structures or phosphorylated neurofilament-positive lesions, which are characteristic of ALS-FUS and frontotemporal lobar degeneration with FUS pathology (FTLD-FUS), were observed in the frontotemporal cortex, motor cortex, medulla oblongata, or spinal cord in either case. Additionally, both cases lacked α-synuclein-positive lesions, argyrophilic grains, tufted astrocytes, and astrocytic plaques in the preferentially affected regions, including brainstem nuclei, striatum, limbic areas, and the frontal cortex. Alzheimer's disease-related neuropathology was minimal in both cases: the Braak stages for neurofibrillary tangle pathology were stage II and stage I, respectively, and amyloid beta (Aβ) deposits with Thal phase 1 were observed only in case 1 (IV-1). No annexin A11-positive cells associated with FTLD-TDP Type C were found in the motor cortex or spinal cord of either case [4].

**Fig. 3:**
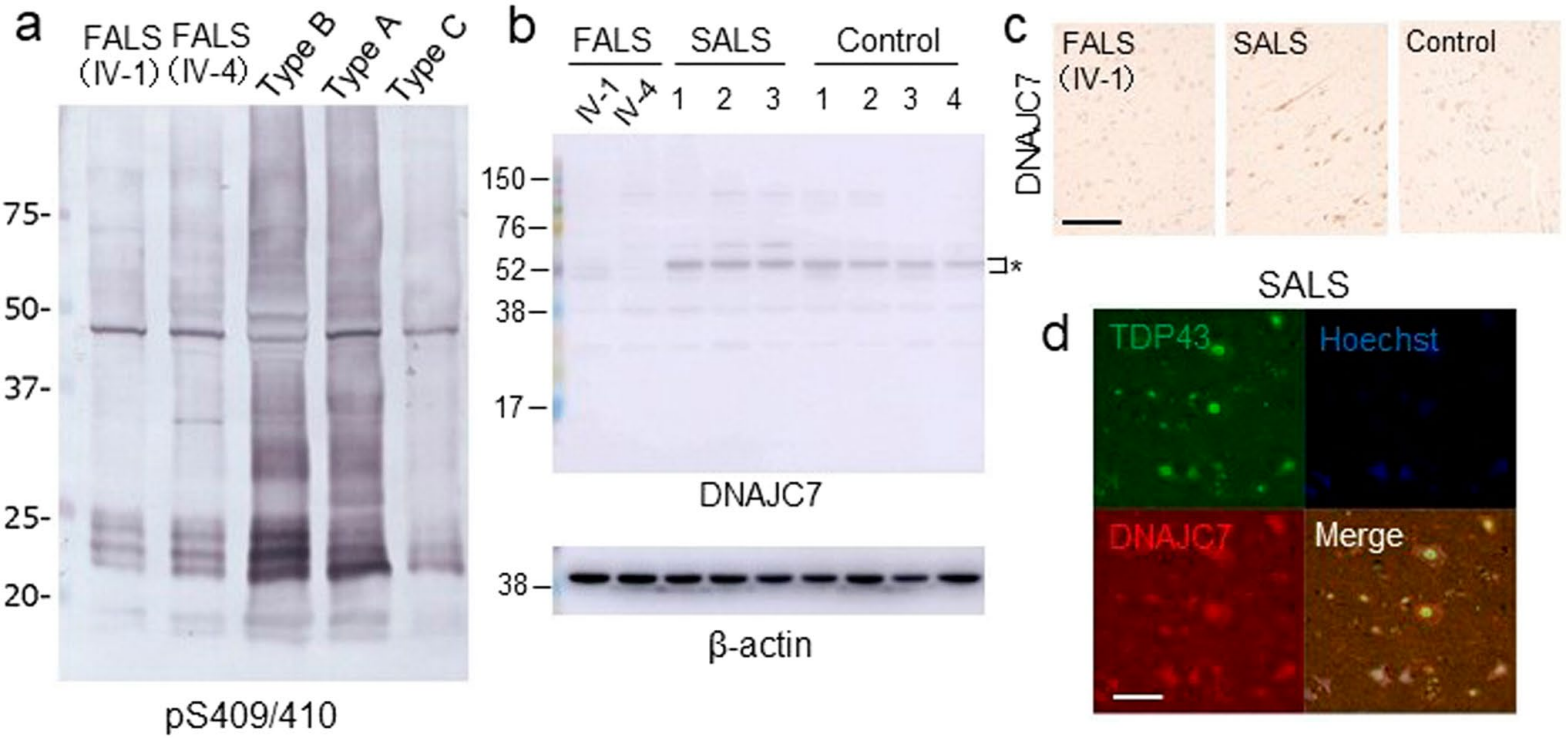
Reduced expression of DNAJC7 protein in the motor cortex of FALS cases with the *DNAJC7* variant. **a** Brain sarkosyl-insoluble fractions from patients with FALS (IV-1, IV-4) and patients with FTLD of various subtypes were subjected to immunoblot analysis using a phosphorylated-TDP43 (Ser409/410) antibody. The immunoblot revealed a distinct pattern of phosphorylated TDP-43, including a prominent band at approximately 50 kDa corresponding to full-length TDP-43, and C-terminal fragments of approximately 23–25 kDa, consistent with the type B pattern previously described [24]. **b** Representative immunoblot showing DNAJC7 protein levels in motor cortex lysates from two FALS patients (IV-1 and IV-4), two SALS patients, and four controls. β-actin was used as the internal loading control. The asterisk (*) indicates the specific band corresponding to DNAJC7. Notably, DNAJC7 protein was barely detectable in the two FALS cases. **c** Immunohistochemical staining of brain sections from a patient with FALS (IV-1), a patient with SALS, and a control individual using an anti-*DNAJC7* antibody. Scale bar: 50 µm. **d** Representative images of double -fluorescent staining of SALS brain sections with anti-TDP-43 and anti-*DNAJC7* antibodies. Scale bar: 20 µm. SALS, sporadic amyotrophic lateral sclerosis; FALS, familial amyotrophic lateral sclerosis; FTLD, frontotemporal lobar degeneration; TDP-43, TAR DNA-binding protein of 43 kDa

Immunoblot analysis of the sarkosyl-insoluble fractions revealed a type B band pattern of phosphorylated TDP-43 C-terminal fragments in the motor cortex, consistent with the results of phosphorylated TDP-43 immunohistochemistry (Fig. 3a). To evaluate the protein expression levels of DNAJC7, we performed Western blot analysis using total brain lysates. As shown in Fig. 3b, DNAJC7 protein levels were markedly reduced in the FALS cases compared with sporadic ALS (SALS) and control cases, confirming a disease-specific downregulation at the protein level. This reduction was also observed by immunohistochemistry, in which DNAJC7 immunoreactivity was substantially diminished in the FALS case (IV-1), whereas *DNAJC7* expression was predominantly nuclear but diffusely distributed in the cytoplasm in the motor cortex of the SALS case (Fig. 3c, d).

### RNA sequencing analysis of patients with *DNAJC7* variants

**Fig. 4:**
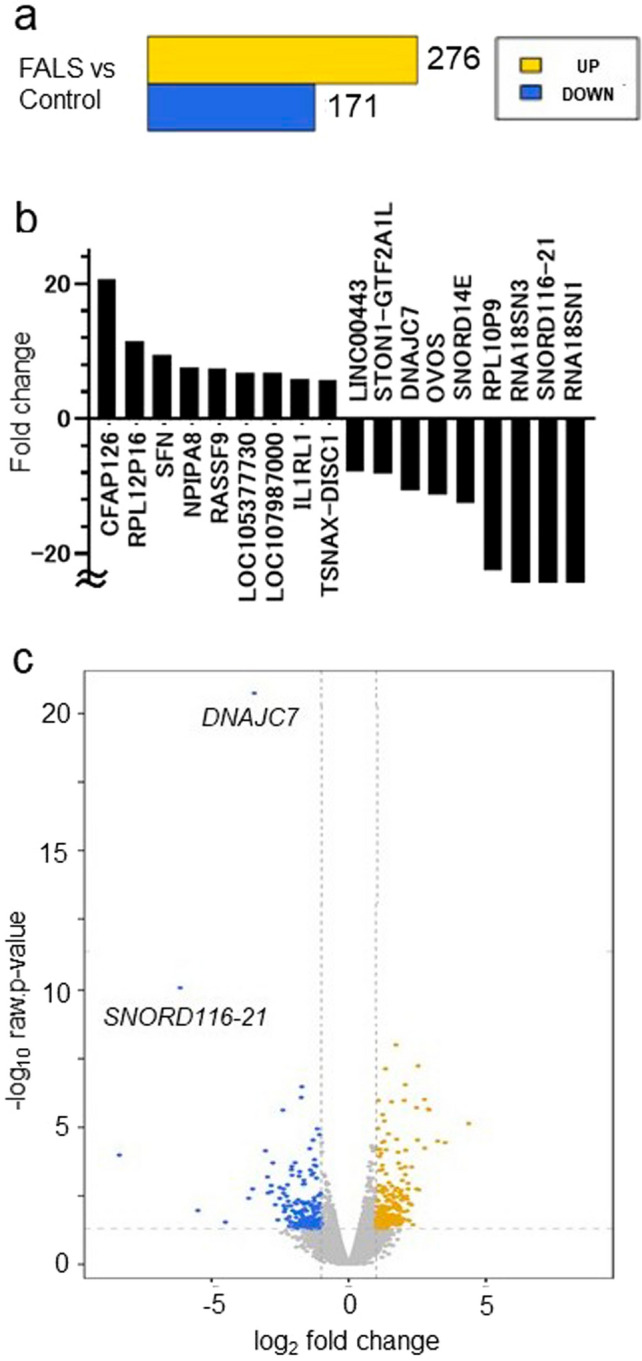
RNA-seq analysis revealed that *DNAJC7* expression was decreased in the motor cortex of the FALS cases with the *DNAJC7* variant. **a** Bar graph showing the number of genes that were upregulated or downregulated in patients with FALS (IV-1, IV-4) compared to normal controls. **b** Waterfall plot depicting the top ten upregulated and downregulated genes in patients with FALS. **c** Volcano plot illustrating the statistical significance against the fold change for gene expression in patients with FALS. FALS, familial amyotrophic lateral sclerosis; RNA-seq, RNA sequencing

We compared mRNA expression in the motor cortex between two patients with the *DNAJC7* variant and four disease control participants using RNA sequencing (RNA-seq). A total of 276 genes were found to be upregulated in the FALS family compared with controls, whereas 171 genes were downregulated (Fig. 4a, ❘FC❘ > 2, raw *p* < 0.05). Notably, *DNAJC7* expression was significantly reduced, both in terms of fold change (FC) and raw *p* value (Fig. 4b, c, FC, -10.67; Log2 FC, -3.42; raw *p* value, 1.56E-21). Furthermore, expression levels of genes in the HSP70 family, particularly *HSPA1A*, *HSPA1B*, and *HSPA6*, showed a trend toward decreased expression in the two patients with FALS compared to the average FPKM values of the disease controls (Supplementary Table 3). To evaluate whether these transcriptomic changes were reflected at the protein level, we performed Western blot analysis using patient brain tissue. While the anti-HSPA1A/B antibody did not yield reliable results, we successfully detected HSPA6 and HSPA8. Consistent with the RNA-seq data, both proteins were decreased in the FALS cases (Supplementary Fig. 3).

### DNAJC7 protein was not expressed when c.518dupC variant was introduced

We transfected HEK293T cells with N-terminal Myc-tagged DNAJC7 expression vectors, both wild-type and with the c.518dupC variant (Supplementary Fig. 2a). Co-transfection with a pCMV-EGFP-IRES-Puro expression vector confirmed comparable transfection efficiency between constructs. Quantification of EGFP-positive cells showed similar fluorescence-positive ratios between the DNAJC7 wild-type group (mean ± SD: 67.4% ± 15.4%) and the mutant group (mean ± SD: 65.6% ± 25.6%) (Supplementary Fig. 2b). However, immunoblot analysis using both anti-DNAJC7 (N-terminal) and anti-Myc antibodies demonstrated that the mutant DNAJC7 protein was undetectable (Supplementary Fig. 2d).

### *HSPA1*, *HSPB1*, *BAG2*, and *DNAJC7* promote TDP-43 condensates disassembly

**Fig. 5:**
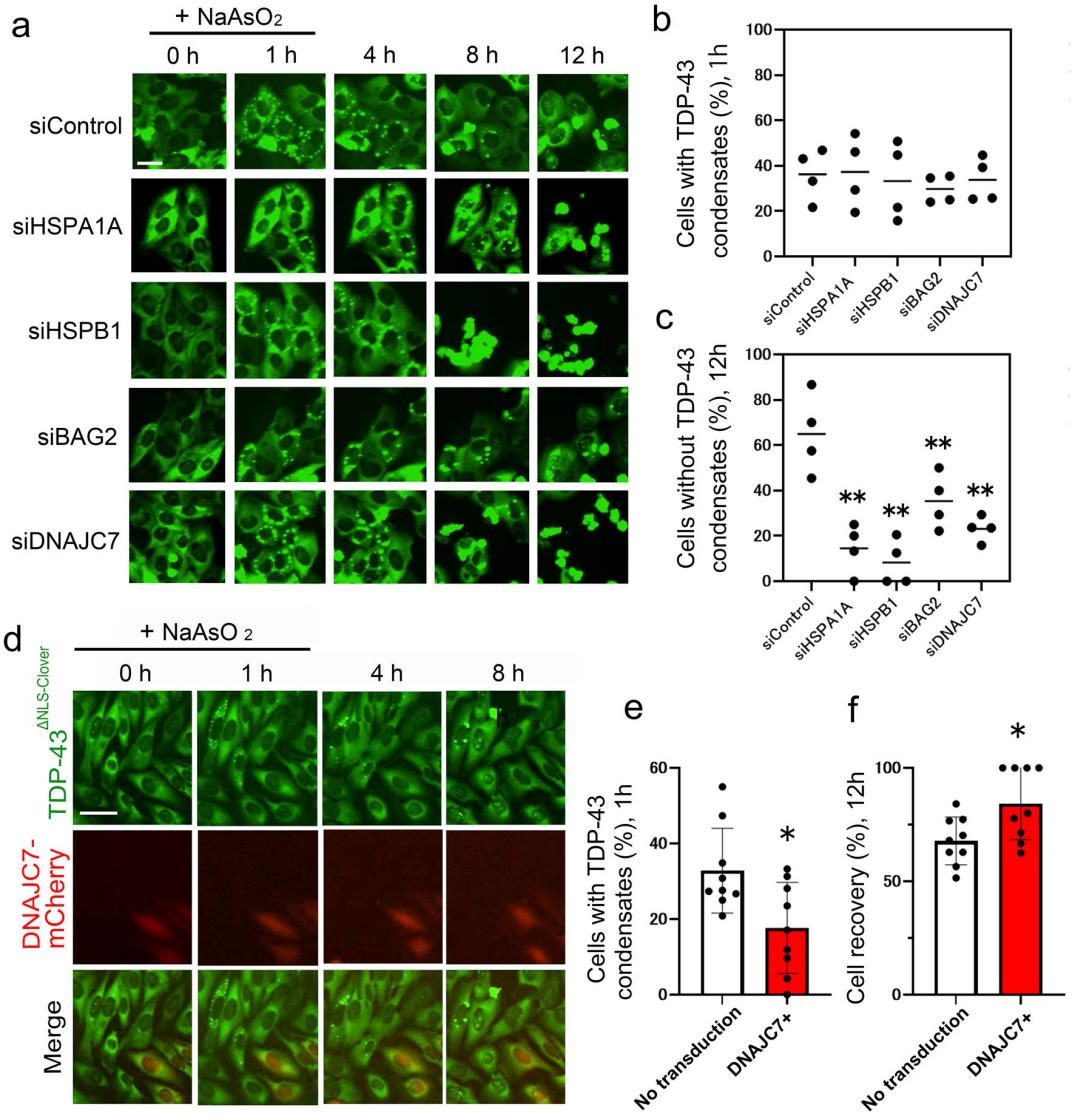
*DNAJC7* is required for the disassembly of stress-induced TDP-43 condensates. **a** Representative living-cell images showing TDP-43^ΔNLS-Clover^ condensates induced by arsenite treatment for 1 h, followed by the removal of stress in U2OS cells treated with control siRNA, or siRNAs targeting HSPA1A, HSPB1, BAG2, and *DNAJC7*. **b** Percentage of cells forming TDP-43 condensates after the 1-h arsenite treatment. Data represents means from four independent experiments with individual values shown. **c** Percentage of cells in which TDP-43 condensates disassemble after 12 h of stress removal. Data represents means from four independent experiments with individual values shown. **p < 0.01 (non-repeated measures analysis of variance and Bonferroni post hoc test). **d** Representative live-cell images showing TDP-43^ΔNLS-Clover^ condensates induced by arsenite treatment for 1 h, followed by stress removal in U2OS cells transduced with *DNAJC7*-mCherry lentivirus to increase *DNAJC7* levels. **e** Percentage of cells forming TDP-43 condensates after 1-h arsenite treatment. Data are shown as mean ± SD from two independent experiments. *p < 0.05 (two-tailed unpaired Student’s *t*-test). **f** Percentage of cells recovering and disassembling TDP-43 condensates after 12 h of stress removal. Data are shown as mean ± SD from two independent experiments. *p < 0.05 (two-tailed unpaired Student’s *t*-test)

TDP-43 is known to undergo liquid–liquid phase separation following oxidative stress or arsenite exposure. Stress-induced assemblies of RNA-binding proteins, such as stress granules, typically dissolve once the stressor [6, 27] is removed. In accordance with a previous study [36], we performed live-cell imaging analysis. Western blotting confirmed that endogenous TDP-43 was constitutively expressed in the stable cell line, and that Dox treatment induced additional expression of the exogenous cytoplasmic TDP-43ΔNLS-Clover (Supplementary Fig. 5). The analysis revealed that cytoplasmic TDP-43 phase-separated condensates, induced by 1-h arsenite treatment, dissolved within 11 h after arsenite removal in approximately 60% of the cells (Fig. 5a). Cells that failed to disassemble the TDP-43 condensates could not recover, exhibiting shrinkage of the cell body, which ultimately led to detachment from the dish. To assess whether depletion of *HSPA1* (a member of the HSP70 family), *HSPB1* (a small heat shock protein), *BAG2* (a co-chaperone involved in the HSP70/HSP90 pathway), and *DNAJC7* (a member of the HSP40 family) impacted the disassembly of arsenite-induced TDP-43 condensates, we pre-treated cells with small interfering RNA (siRNA) targeting the mRNA encoding each protein 72 h before arsenite exposure. Effective knockdown of each target genes was confirmed by RT-PCR (Supplementary Fig. 4). Knockdown of *HSPA1*, *HSPB1*, *BAG2*, and *DNAJC7* did not affect the formation of arsenite-induced TDP-43 condensates (Fig. 5a, b). However, the knockdown of corresponding genes significantly inhibited the disassembly of the condensates after arsenite removal (Fig. 5a, c, **p < 0.01).

Next, we tested whether the overexpression of *DNAJC7* promotes the disassembly of arsenite-induced TDP-43 condensates. Cells were infected with a lentiviral vector encoding *DNAJC7*-mCherry 72 h prior to arsenite exposure. One hour after arsenite treatment, the number of cells with cytoplasmic TDP-43 condensates was compared between mCherry-negative and mCherry-positive cells. Overexpression of DNAJC7 significantly suppressed the number of cells with arsenite-induced cytoplasmic TDP-43 condensates (Fig. 5d, e, *p < 0.05). We also evaluated cell recovery at 12 h after arsenite exposure and found that DNAJC7 overexpression significantly enhanced recovery (Fig. 5f, *p < 0.05). These results suggest that DNAJC7 overexpression at least suppresses the formation of TDP-43-containing condensates, which may contribute to improved cellular resilience following stress.

### Loss of *dnajc7* is associated with increased TDP-43 aggregation

**Fig. 6:**
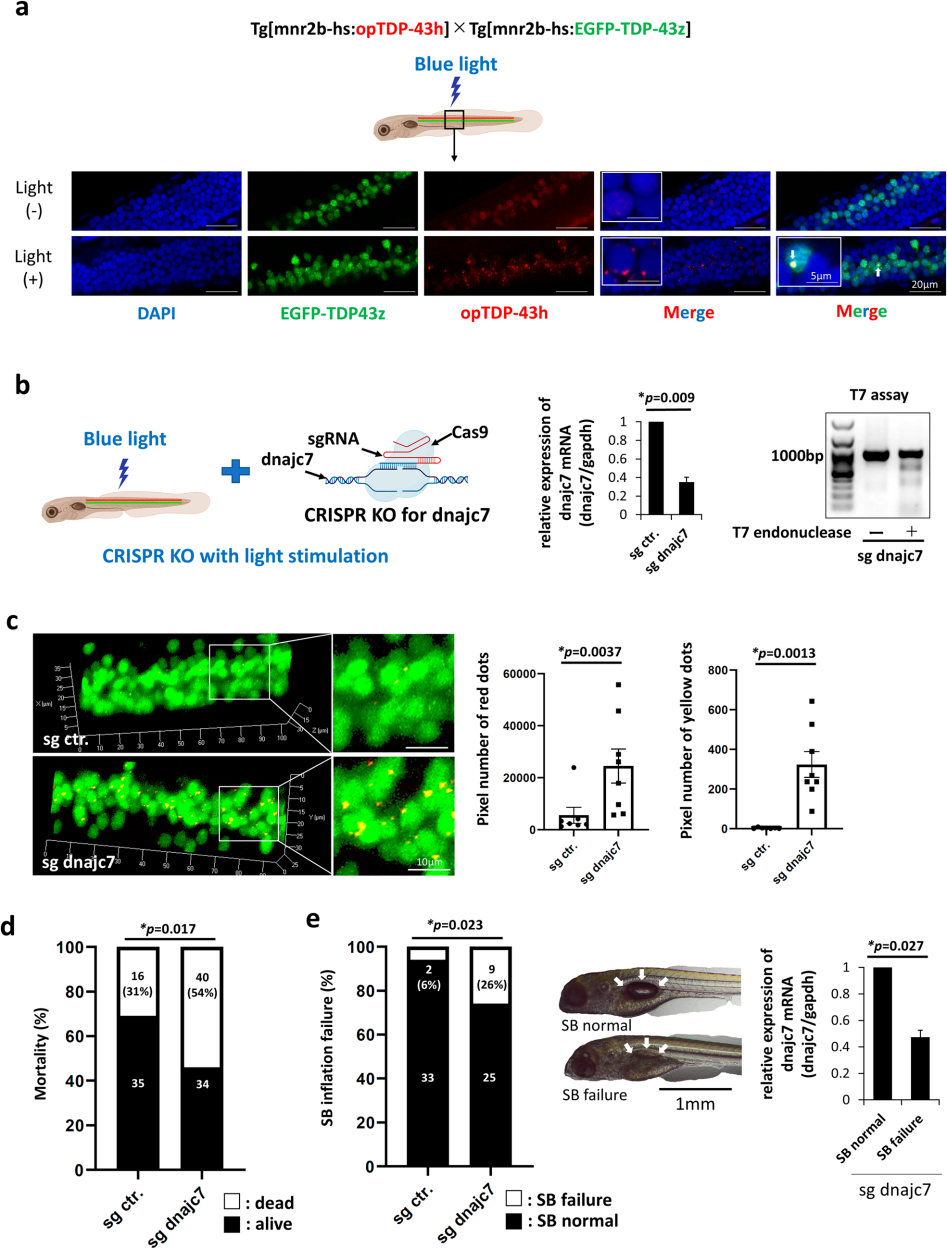
Knockdown of *dnajc7* leads to impaired neurodevelopment through accelerated TDP-43 aggregation. **a** Schematic representation and representative image of optogenetically modified transgenic zebrafish Tg[mnr2b-hs:opTDP-43h] Tg[mnr2b-hs:EGFP-TDP-43z]. The opTDP-43h construct encodes a human TDP-43 anchoring optic module, cryptochrome (CRY2), and RFP. Under basal conditions, the red signal is weakly expressed in the nucleus. After blue light illumination for 48–96 h, a clear aggregation of TDP-43 is observed at perinuclear sites. EGFP-TDP-43z encodes zebrafish TDP-43, which is recruited to and aggregated at the perinuclear site upon light-induced aggregation of opTDP-43 (yellow foci indicated by white arrows). **b** (Left) Schematic representation of CRISPR-Cas9-mediated *dnajc7* knockout in zebrafish. (Middle) Quantitative reverse transcription PCR analysis of *dnajc7* expression levels in sg ctr and sg dnajc7 injected embryos at 6 dpf (days post-fertilization). (Right) Agarose gel image showing the T7 endonuclease I assay results for the sgRNAs targeting *dnajc7*, confirming successful knockout. **c** (Left) Representative microscopic images showing TDP-43 aggregates in sg ctr and sg *dnajc7* injected ALS zebrafish. (Right) Quantification of pixel count for TDP-43 aggregates in sg ctr (7 fields) and sg dnajc7 (8 fields) injected ALS zebrafish at 6 dpf. Data are presented as mean ± SD from one of three independent experiments. Statistical differences were calculated using the Mann–Whitney U-test. **d** Fraction of dead embryos in sg ctr and sg dnajc7 injected ALS zebrafish at 6 dpf. Data show the number of dead or survived embryos from one of three independent experiments. Differences were calculated using Fisher’s exact test. **e** (Left) Fraction of embryos with failed SB inflation in sg ctr and sg dnajc7 injected ALS zebrafish at 6 dpf. (Middle) Representative images of SB in the embryos. (Right) Quantitative reverse transcription PCR analysis of *dnajc7* expression in the survived sg dnajc7 injected embryos at 6dpf. Embryos with SB inflation failure showed significantly lower dnajc7 mRNA expression than those with normal SB inflation. Data show mean ± SD from one of three independent experiments. Statistical differences were calculated using Fisher's exact test or Mann–Whitney U-test. SB, swim bladder; ALS, amyotrophic lateral sclerosis; TDP-43, TAR DNA-binding protein of 43 kDa

To further investigate the role of *DNAJC7* in FALS in vivo, we utilized a zebrafish model harboring double transgenes, Tg[mnr2b-hs:opTDP-43h]Tg[mnr2b-hs:EGFP-TDP43z] [5]. This model enables the motor neuron-specific recapitulation of TDP-43 aggregation through temporal light stimulation (Fig. 6a). The RFP-tagged human TDP-43 (opTDP-43h) is anchored by an optogenetic module derived from *Arabidopsis thaliana* (cryptochrome: CRY2), which undergoes conformational changes upon exposure to optical energy, resulting in entanglement and aggregation at the perinuclear site (red foci). Following the aggregation of opTDP-43h, zebrafish TDP-43 without the optogenetic module (EGFP-TDP-43z) is recruited and mislocalised into cytoplasmic inclusions (green foci), forming perinuclear yellow foci (white arrows, Fig. 6a) [5, 38].

At the outset, we microinjected sgRNAs targeting *dnajc7* together with Cas9 protein into single-cell embryos of this transgenic zebrafish model (Fig. 6b, left), successfully knocking out *dnajc7*, as evidenced by decreased *dnajc7* mRNA expression (Fig. 6b, middle). DNA cleavage was confirmed by T7 endonuclease I assay (Fig. 6b, right). To assess whether the loss of *dnajc7* influences TDP-43 aggregation, we applied light stimulation for 144 h to the *dnajc7*-depleted ALS zebrafish, followed by microscopic analysis. Notably, we observed significant TDP-43 aggregation in motor neurons of the *dnajc7* knockout zebrafish (sg dnajc7) compared to control sgRNA-treated zebrafish (sg ctr.) (Fig. 6c). The increased TDP-43 aggregation comprised both opTDP-43h-derived foci (red) and recruited EGFP-TDP-43 (yellow, indicating co-localisation of green and red signals), primarily localised at the perinuclear site (Fig. 6c). Quantitative analysis confirmed that these exacerbated TDP-43 aggregations were statistically significant for both red and yellow signals (Fig. 6c) suggesting that *dnajc7* knockout enhanced TDP-43 aggregation in ALS zebrafish models.

### High mortality and impaired neurodevelopment in *dnajc7*-deleted zebrafish

Next, whether the loss of *dnajc7* affects the phenotypes of the ALS zebrafish model was examined. Notably, *dnajc7* depletion resulted in higher mortality and worsened swim bladder (SB) inflation failure (Fig. 6d, e), a key phenotype indicating impaired neurodevelopment, particularly of motor neurons [5, 12]. Moreover, among the surviving embryos from the sg dnajc7 injection group at 6 days post-fertilization (dpf) (Fig. 6d, right bar, black), embryos with SB inflation failure showed lower *dnajc7* mRNA expression levels compared to those with normal SB inflation (Fig. 6e). These findings suggest that decreased *dnajc7* expression is associated with impaired neurodevelopment, likely through the exacerbation of TDP-43 aggregation in motor neurons.

## Discussion

Here, we report three siblings with ALS from a single family*.* All three patients initially presented with muscle weakness in the lower or upper limbs, followed by progressive muscle weakness, limb atrophy, bulbar palsy, and respiratory failure. Two of the three patients died approximately 8 years after disease onset and underwent autopsy. Pathological examination revealed phosphorylated TDP-43 positive neuronal cytoplasmic inclusions in the motor neurons and frontal and temporal cortices. Immunoblot analysis confirmed a type B pattern of phosphorylated TDP-43 protein in the brain tissue of precentral gyrus. These pathological findings are indistinguishable from those observed in typical sporadic ALS.

We identified a homozygous frameshift variant in *DNAJC7* in the family. Immunohistochemical and RNA-seq analyses indicated that the expression of *DNAJC7* at both the protein and RNA levels was substantially decreased in the affected brain tissues (Figs. 3, 4). In 2019, a large exome-wide association study revealed that the loss-of-function variants in *DNAJC7* are significantly associated with ALS in mainly European population [18]. Subsequent studies in Chinese, Taiwanese, Japanese, and Italian populations generally supported the results [18, 25, 28, 45, 47, 51, 52], although the carrier frequency is low (0–0.52%). Although more than 10,000 patients with ALS have been screened, no cases with biallelic loss-of-function variants in *DNAJC7*, with family history, or with pathological examination have been reported to date (Supplementary Table 4). There are potentially multiple heterozygous carriers within this family; however, no other relatives besides the three affected siblings have been reported to develop ALS. Our finding suggests that heterozygous variants in *DNAJC7* behave as a genetic risk factor, whereas biallelic variants behave as Mendelian variants. Similar situations have been reported in neurodegenerative diseases such as *COQ2* in multiple system atrophy [46].

We also report that the loss-of-function of *DNAJC7* leads to decreased disassembly of TDP-43 following arsenite administration in cultured cells. In a zebrafish model, knockout of *dnajc7* led to increased TDP-43 aggregation in motor neurons and reduced survival, supporting the notion that loss of DNAJC7 function promotes TDP-43 pathology. As part of our phenotypic analysis, we assessed swim bladder (SB) inflation failure, which has been previously shown to be linked to motor neuron development. This decision was based on findings by Asakawa et al. [5], who demonstrated that zebrafish expressing mutant TDP-43 (A315T) exhibited SB failure. Given the similarities in TDP-43 pathology, we adopted SB inflation as a phenotypic marker in our study. Additionally, recent research has shown that SB inflation depends on proper motor neuron function and swim-up behaviour [11]. For example, knockout of *sox2* leads to downregulation of axon guidance genes such as *sema3bl*, *ntn1b*, and *robo2*, resulting in disrupted motor neuron development, impaired swim-up behaviour, and subsequent SB inflation failure. These findings collectively support the validity of using SB inflation as a functional surrogate for motor neuron integrity in zebrafish.

*DNAJC7* is a DNAJ (HSP40) co-chaperone with a characteristic J domain that interacts with HSP70, promoting its hydrolysis of ATP and increasing the HSP70 substrate protein interaction to aid folding. *DNAJC7* itself can bind the prion-like domain of TDP-43 [13]. Meanwhile, HSP70 (HSPA) can also bind the prion-like domain of TDP-43, promoting its refolding and clearance [32]. Therefore, *DNAJC7* refolds TDP-43 as a soluble state via HSP70 or directly. The coexistence of *DNAJC7* and HSP70 in TDP-43 positive inclusion bodies may help maintain their liquid nature by avoiding their transition to amyloid aggregate formation [22]. Interestingly, the RNA-seq data revealed reduced expression of not only *DNAJC7* but also the HSP70 family, including *HSAPA1A*, *HSAPA1B*, and *HSAPA6* in the brain, which is consistent with a previous report showing HSP70 expression was decreased in sporadic ALS patient spinal cord [14]. These results suggest that *DNAJC7* dysfunction, coupled with aging, disrupts the chaperone sub-network leading to a collapse in proteostasis primarily affecting TDP-43. This perturbation ultimately results in motor neuronal death [9].

Overexpression of HSP70 suppresses cytoplasmic TDP-43 aggregate formation in vitro study [33], indicating that chaperone proteins such as HSP70 are promising therapeutic targets for ALS. In this study, we showed that overexpression of *DNAJC7* promotes the disassembly of arsenite-induced TDP-43 condensates in a U2OS cell model (Fig. 5d, e), suggesting that overexpressed *DNAJC7* may attenuate ALS pathophysiology by controlling the misfolded TDP-43. Another co-chaperone, DNAJB2a, has been reported to play an important role in TDP-43 clearance in an in vitro study [14]. DNAJB2a or HSPB1 overexpression in motor neurons of SOD1 G93A transgenic mice ameliorated disease progression [39, 43]. Therefore, not only HSP70 but also co-chaperones have to be included in the potential therapeutic target linking TDP-43 pathology.

Taken together, although the number of cases is limited, the present paper suggests that biallelic loss-of-function variants in *DNAJC7* cause a familial form of ALS whose clinical and pathological phenotype is indistinguishable from that of sporadic ALS, and emphasizes *DNAJC7* as a promising therapeutic target for ALS.

## Supplementary Information

Below is the link to the electronic supplementary material.

Supplementary file1 (TIFF 9093 KB)

Supplementary file2 (JPG 92 KB)

Supplementary file3 (JPG 56 KB)

Supplementary file4 (JPG 93 KB)

Supplementary file5 (JPG 16 KB)

Supplementary file6 (DOCX 44 KB)

## Data Availability

The data supporting the findings of this study are available from the corresponding authors upon reasonable request.
