# Supplementary figures and images for: Biallelic variants in DNAJC7 cause familial amyotrophic lateral sclerosis with the TDP-43 pathology
Source: Acta Neuropathol. 2025 Aug 13;150(1):19. doi: 10.1007/s00401-025-02899-y (PMC12350594; doi:10.1007/s00401-025-02899-y)

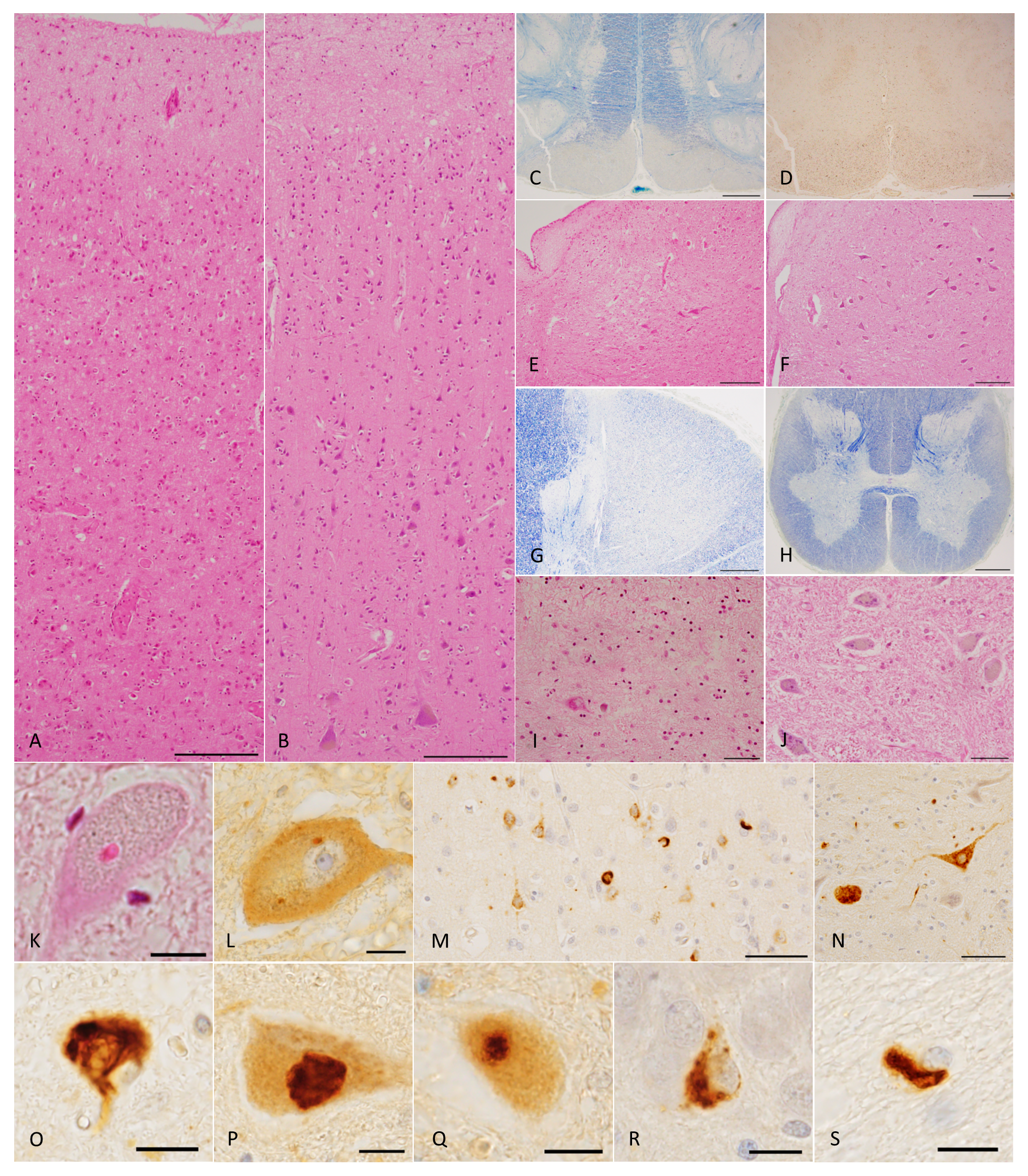

Supplement: Supplementary file 1 — Supplementary file1 (TIFF 9093 KB) [file 401_2025_2899_MOESM1_ESM.tif]

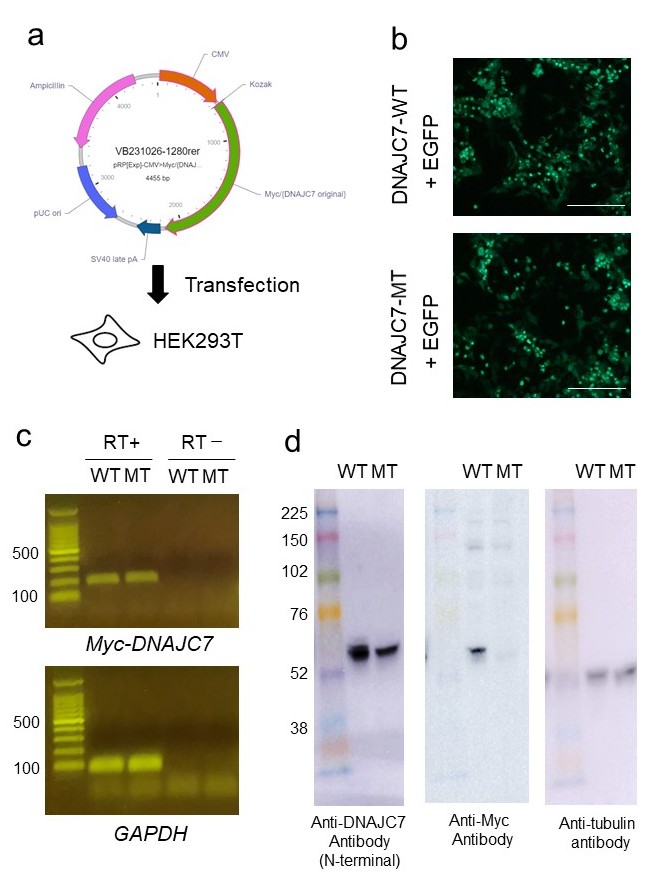

Supplement: Supplementary file 2 — Supplementary file2 (JPG 92 KB) [file 401_2025_2899_MOESM2_ESM.jpg]

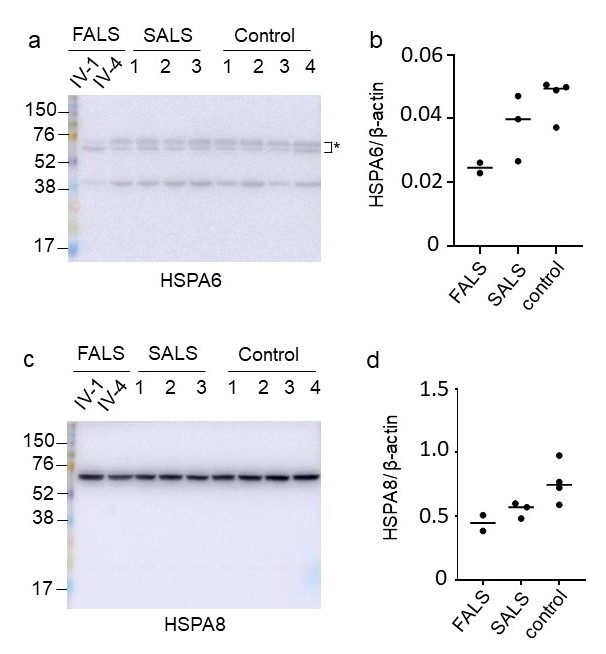

Supplement: Supplementary file 3 — Supplementary file3 (JPG 56 KB) [file 401_2025_2899_MOESM3_ESM.jpg]

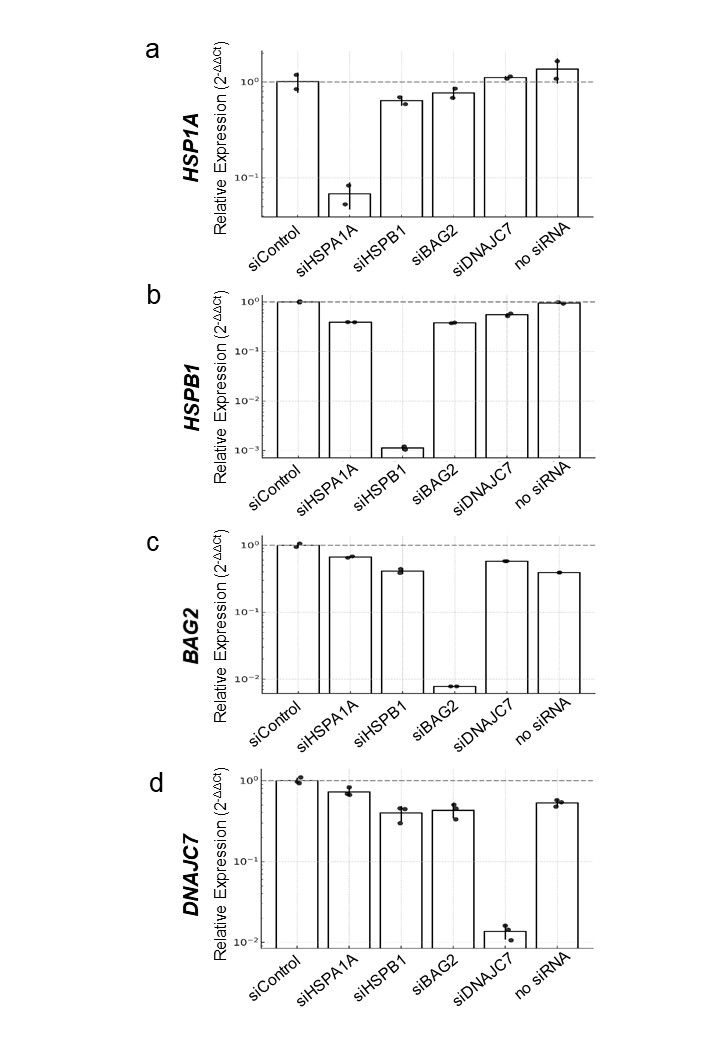

Supplement: Supplementary file 4 — Supplementary file4 (JPG 93 KB) [file 401_2025_2899_MOESM4_ESM.jpg]

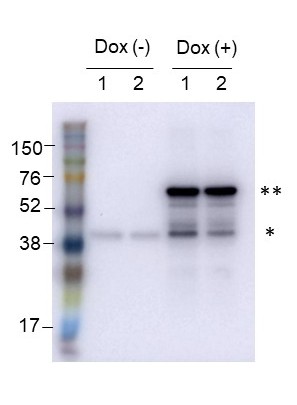

Supplement: Supplementary file 5 — Supplementary file5 (JPG 16 KB) [file 401_2025_2899_MOESM5_ESM.jpg]
