# Supplementary material for: Biallelic variants in DNAJC7 cause familial amyotrophic lateral sclerosis with the TDP-43 pathology
Source: Acta Neuropathol. 2025 Aug 13;150(1):19. doi: 10.1007/s00401-025-02899-y (PMC12350594; doi:10.1007/s00401-025-02899-y)
Supplement: Supplementary file 6 — Supplementary file6 (DOCX 44 KB) [file 401_2025_2899_MOESM6_ESM.docx]

**Supplementary information**

**Supplementary Fig. 1. Phosphorylated TDP-43 pathology, Bunina bodies, and severe loss of the upper and lower motor neurons in the older sister (IV-4) with a homozygous c.518dupC frameshift variant in *DNAJC7*.**

1. Severe neuronal loss with glial proliferation and tissue rarefaction in all cortical layers of the primary motor cortex. No Betz cell was noted. H&E staining. Scale bar: 200 μm.
2. Primary motor cortex in a 68-year-old pathologically normal control case. Betz cells in layer V are noted, and the structure of the cortex is spared. H&E staining. Scale bar: 200 μm.

(c, d) Severe loss of myelin (c) with gliosis (d) in the pyramidal tract in the medulla oblongata. (c) Klüver–Barrera staining and (d) Iba1 immunohistochemistry showing microglial activation. Scale bars: 1 mm.

(e) Severe neuronal loss with gliosis and tissue rarefaction in the hypoglossal nucleus. H&E staining. Scale bar: 200 μm.

(f) Hypoglossal nucleus in a 68-year-old pathologically normal control case. Many motor neurons are observed, and tissue rarefaction is absent. H&E staining. Scale bar: 200 μm.

(g) Severe degeneration in the lateral tract, with myelin loss in the cervical cord. The dorsal spinocerebellar tract remains relatively spared. Klüver–Barrera staining. Scale bar: 500 μm.

(h) Degeneration in the lateral tract and severe loss of anterior horn cells in the lumbar cord. Klüver–Barrera staining. Scale bar: 1 mm.

(i) Severe loss of neurons with gliosis and tissue rarefaction in the lumbar anterior horn. H&E staining. Scale bar: 50 μm.

(j) Spinal anterior horn cells in a 74-year-old pathologically normal control case. Neither glial proliferation nor tissue rarefaction was noted. H&E staining. Scale bar: 50 μm.

(k,l) Bunina bodies in the lumbar anterior horn. (k) H&E staining and (l) cystatin C immunohistochemistry. Scale bars: 10 μm.

(m) Phosphorylated TDP-43-positive NCIs in the primary motor cortex. No dystrophic neurite was observed. pS409/410-2 immunohistochemistry. Scale bar: 50 μm.

(n) Phosphorylated TDP-43-positive inclusions in the hypoglossal nucleus. Skein-like (left) and diffuse granular structures (right) are seen. pS409/410-2 immunohistochemistry. Scale bar: 50 μm.

(o-q) (o,p) Phosphorylated TDP-43-positive skein-like NCIs and (q) intranuclear inclusion in the lumbar anterior horn. pS409/410-2 immunohistochemistry. Scale bars: 10 μm.

(r) Phosphorylated TDP-43-positive inclusions in the hippocampal dentate gyrus. pS409/410-2 immunohistochemistry. Scale bar: 10 μm.

(s) A phosphorylated TDP-43-positive coiled body-like glial inclusion in the white matter of the temporal lobe. pS409/410-2 immunohistochemistry. Scale bar: 10 μm.

(b, f, and j) Control cases presented lacked any neurodegenerative changes except for minimal NFTs. H&E, hematoxylin-eosin; NCI, neuronal cytoplasmic inclusions; NFTs, neurofibrillary tangles; TDP-43, TAR DNA-binding protein of 43 kDa

**Supplementary Fig. 2. Expression of wild-type and mutant DNAJC7 constructs in HEK293T cells.**

(a) Schematic diagram of the expression vector containing the Myc-tagged DNAJC7 wild-type (WT) or c.518dupC mutant (MT) constructs under the control of the CMV promoter, used for transfection into HEK293FT cells.

(b) Representative fluorescence microscopy images showing HEK293T cells co-transfected with the pCMV-EGFP-IRES-Puro expression vector and either the DNAJC7-WT or DNAJC7-MT expression vector. Scale bars = 200 μm.

(c) RT-PCR analysis of *Myc*-*DNAJC7* and *GAPDH* mRNA expression in HEK293T cells transfected with WT or MT constructs. Both WT and MT constructs showed comparable levels of *Myc*-*DNAJC7* transcript expression. RT+, with reverse transcriptase; RT–, without reverse transcriptase (negative control).

(d) Immunoblot analysis of cell lysates from HEK293T cells transfected with DNAJC7-WT or MT using antibodies against DNAJC7 (N-terminal), Myc, and α-tubulin (loading control). While both constructs showed similar expression when detected with the N-terminal DNAJC7 antibody, reduced signal was observed for the mutant construct with the anti-Myc antibody.

**Supplementary Fig. 3. Expression levels of HSPA6 and HSPA8 in the motor cortex of individuals with familial ALS (FALS), sporadic ALS (SALS), and controls.**

(a) Representative immunoblot of HSPA6 protein in motor cortex lysates from two FALS patients (IV-1 and IV-4), three SALS patients, and four controls. β-actin was used as the internal loading control. The asterisk (*) indicates the specific band corresponding to HSPA6.

(b) Quantification of HSPA6 protein levels normalized to β-actin. Data represent means with individual values shown.

(c) Representative immunoblot of HSPA8 protein using the same samples as in (a).

(d) Quantification of HSPA8 protein levels normalized to β-actin. Data represent means with individual values shown.

**Supplementary Fig. 4. Validation of siRNA-mediated knockdown efficiency for *HSPA1A*, *HSPB1*, *BAG2*, and *DNAJC7* in HEK293T cells.**

Relative mRNA expression levels of (a) *HSPA1A*, (b) *HSPB1*, (c) *BAG2*, and (d) *DNAJC7* were measured by quantitative real-time PCR using the ΔΔCt method. U2OS Cells were transfected with siRNAs targeting *HSPA1A* (siHSPA1A), *HSPB1* (siHSPB1), *BAG2* (siBAG2), *DNAJC7* (siDNAJC7), a non-targeting control siRNA (siControl), or treated with lipofectamine only (no siRNA). Data show mean ± SD with individual values.

**Supplementary Fig. 5. Expression of doxycycline-inducible TDP-43ΔNLS-Clover and endogenous TDP-43 in U2OS cells.**

Stable U2OS cells were cultured in 6-well plates and treated with or without doxycycline (Dox; 100 ng/mL) for 24 hours to induce expression of TDP-43ΔNLS-Clover. Total TDP-43 protein levels were analysed by Western blotting. Two technical replicates (lanes 1 and 2) were performed for each condition, with or without doxycycline (DOX). In Dox-treated cells (Dox [+]), a marked increase in TDP-43 signal was observed corresponding to both endogenous TDP-43 (*) and exogenously expressed TDP-43ΔNLS-Clover (**). In the absence of doxycycline (Dox [−]), only endogenous TDP-43 was detected, with no expression of the exogenous fusion protein.

**Supplementary Table 1** Plasmid information

| Name | Backbone | Source or Reference | Additional information |
| --- | --- | --- | --- |
| pTRE3G-TDP-43 ^∆NLS(K82A, R83A,K84A)-Clover^ | Lentiviral vector | The sequence of TDP-43 ∆NLS (K82A, R83A, K84A) is adopted from Gasset-Rosa, F. et al. Neuron 102, 339-357 (2019). |  |
| pTRE3G-hDNAJC7-mCherry | Lentiviral vector | The present study |  |
| pCMV-Myc-hDNAJC7 | Mammalian gene expression vector | The present study | Myc-tag |
| pCMV-Myc-hDNAJC7 with mutation (c.518dupC) | Mammalian gene expression vector | The present study | Myc-tag |
| pCMV-EGFP-IRES-Puro | Mammalian gene expression vector | The present study |  |

**Supplementary Table 2** Rare variants detected in the candidate regions

| **Chromosome** | **Position in hg38** | **Reference** | **Variant** | **Gene** | **Annotation** |
| --- | --- | --- | --- | --- | --- |
| chr17 | 16420147 | G | A | *TRPV2* | c.233G>A, p.R78Q (NM_016113) |
| chr17 | 41731547 | C | T | *HAP1* | c.1015G>A, p.D339N (NM_177977) |
| chr17 | 41990344 | - | G | *DNAJC7* | c.518dupC, p.A174Cfs*27 (NM_003315.4) |
| chr17 | 42180877 | G | T | *KCNH4* | c.69C>A, p.D23E (NM_012285) |
| chr17 | 45034774 | C | T | *DCAKD* | c.112G>A, p.V38I (NM_001288655) |

**Supplementary Table 3** Relative FPKM values of each HSP family mRNA from two FALS cases (IV-1, IV-4) compared to four disease controls

| **Gene_**  **Symbol** | **FPKM_**  **IV-1** | **FPKM_**  **IV-4** | **FPKM_**  **4 controls** | **IV-1/controls** | **IV-4/controls** |
| --- | --- | --- | --- | --- | --- |
| DNAJA1 | 72.70 | 73.03 | 84.65 | 0.86 | 0.86 |
| DNAJA2 | 32.64 | 22.15 | 32.04 | 1.02 | 0.69 |
| DNAJA3 | 16.02 | 11.83 | 14.13 | 1.13 | 0.84 |
| DNAJA4 | 28.25 | 18.21 | 31.79 | 0.89 | 0.57 |
| DNAJB1 | 24.63 | 19.10 | 27.16 | 0.91 | 0.70 |
| DNAJB2 | 52.17 | 48.94 | 49.70 | 1.05 | 0.98 |
| DNAJB4 | 22.64 | 27.19 | 28.00 | 0.81 | 0.97 |
| DNAJB5 | 4.68 | 3.88 | 3.39 | 1.38 | 1.14 |
| DNAJB6 | 61.71 | 53.03 | 62.41 | 0.99 | 0.85 |
| DNAJB9 | 17.88 | 11.12 | 14.39 | 1.24 | 0.77 |
| DNAJB11 | 9.02 | 9.11 | 7.94 | 1.14 | 1.15 |
| DNAJB12 | 6.11 | 7.88 | 6.50 | 0.94 | 1.21 |
| DNAJB14 | 36.67 | 26.65 | 34.99 | 1.05 | 0.76 |
| DNAJC1 | 6.83 | 7.91 | 6.06 | 1.13 | 1.31 |
| DNAJC2 | 6.61 | 5.95 | 6.08 | 1.09 | 0.98 |
| DNAJC3 | 11.11 | 11.00 | 9.64 | 1.15 | 1.14 |
| DNAJC4 | 6.83 | 6.77 | 7.34 | 0.93 | 0.92 |
| DNAJC5 | 29.90 | 23.00 | 29.35 | 1.02 | 0.78 |
| DNAJC6 | 89.01 | 56.74 | 84.57 | 1.05 | 0.67 |
| DNAJC7 | 1.83 | 5.07 | 33.24 | 0.06 | 0.15 |
| DNAJC8 | 49.71 | 38.56 | 47.49 | 1.05 | 0.81 |
| DNAJC9 | 3.71 | 5.57 | 4.47 | 0.83 | 1.25 |
| DNAJC10 | 4.32 | 6.14 | 4.71 | 0.92 | 1.30 |
| DNAJC11 | 10.06 | 9.76 | 10.42 | 0.97 | 0.94 |
| DNAJC12 | 11.38 | 10.24 | 14.36 | 0.79 | 0.71 |
| DNAJC13 | 9.17 | 12.64 | 10.19 | 0.90 | 1.24 |
| DNAJC14 | 6.94 | 6.10 | 6.53 | 1.06 | 0.93 |
| DNAJC15 | 7.66 | 8.48 | 9.08 | 0.84 | 0.93 |
| DNAJC16 | 8.82 | 7.72 | 7.77 | 1.13 | 0.99 |
| DNAJC17 | 2.12 | 2.74 | 2.69 | 0.79 | 1.02 |
| DNAJC18 | 13.07 | 9.16 | 11.83 | 1.10 | 0.77 |
| DNAJC19 | 12.45 | 11.22 | 13.92 | 0.89 | 0.81 |
| DNAJC21 | 6.73 | 7.76 | 7.81 | 0.86 | 0.99 |
| DNAJC24 | 8.67 | 9.02 | 7.76 | 1.12 | 1.16 |
| DNAJC25 | 2.84 | 3.03 | 2.88 | 0.99 | 1.05 |
| DNAJC27 | 6.76 | 5.31 | 6.90 | 0.98 | 0.77 |
| DNAJC28 | 1.96 | 3.75 | 3.16 | 0.62 | 1.18 |
| DNAJC30 | 4.87 | 4.29 | 4.92 | 0.99 | 0.87 |
| DNAJC18 | 13.07 | 9.16 | 11.83 | 1.10 | 0.77 |
| DNAJC19 | 12.45 | 11.22 | 13.92 | 0.89 | 0.81 |
| DNAJC21 | 6.73 | 7.76 | 7.81 | 0.86 | 0.99 |
| DNAJC24 | 8.67 | 9.02 | 7.76 | 1.12 | 1.16 |
| DNAJC25 | 2.84 | 3.03 | 2.88 | 0.99 | 1.05 |
| DNAJC27 | 6.76 | 5.31 | 6.90 | 0.98 | 0.77 |
| DNAJC28 | 1.96 | 3.75 | 3.16 | 0.62 | 1.18 |
| DNAJC30 | 4.87 | 4.29 | 4.92 | 0.99 | 0.87 |
| HSP90AA1 | 391.91 | 326.48 | 465.17 | 0.84 | 0.70 |
| HSP90AB1 | 344.83 | 195.17 | 326.39 | 1.06 | 0.60 |
| HSP90B1 | 84.49 | 75.50 | 82.20 | 1.03 | 0.92 |
| HSPA1A | 47.32 | 52.89 | 89.34 | 0.53 | 0.59 |
| HSPA1B | 44.82 | 44.69 | 85.59 | 0.52 | 0.52 |
| HSPA2 | 34.34 | 28.62 | 37.01 | 0.93 | 0.77 |
| HSPA4 | 28.51 | 24.59 | 31.00 | 0.92 | 0.79 |
| HSPA5 | 39.94 | 37.40 | 41.73 | 0.96 | 0.90 |
| HSPA6 | 0.36 | 0.55 | 1.17 | 0.31 | 0.47 |
| HSPA8 | 497.76 | 406.77 | 556.10 | 0.90 | 0.73 |
| HSPA9 | 51.66 | 42.15 | 51.12 | 1.01 | 0.82 |
| HSPA12A | 35.55 | 29.63 | 39.38 | 0.90 | 0.75 |
| HSPA12B | 1.20 | 1.54 | 1.09 | 1.10 | 1.41 |
| HSPA13 | 38.76 | 26.58 | 35.59 | 1.09 | 0.75 |
| HSPA14 | 6.60 | 4.82 | 4.72 | 1.40 | 1.02 |
| HSPB1 | 79.55 | 73.42 | 63.62 | 1.25 | 1.15 |
| HSPB2 | 2.24 | 1.44 | 2.36 | 0.95 | 0.61 |
| HSPB3 | 4.65 | 1.12 | 3.52 | 1.32 | 0.32 |
| HSPB8 | 44.61 | 37.36 | 31.04 | 1.44 | 1.20 |
| HSPB11 | 13.27 | 13.29 | 13.83 | 0.96 | 0.96 |
| HSPD1 | 50.00 | 45.84 | 53.56 | 0.93 | 0.86 |
| HSPE1 | 32.25 | 41.04 | 38.36 | 0.84 | 1.07 |
| HSPH1 | 50.63 | 53.87 | 63.22 | 0.80 | 0.85 |
| HSPB8 | 44.61 | 37.36 | 31.04 | 1.44 | 1.20 |
| HSPB11 | 13.27 | 13.29 | 13.83 | 0.96 | 0.96 |
| HSPD1 | 50.00 | 45.84 | 53.56 | 0.93 | 0.86 |
| HSPE1 | 32.25 | 41.04 | 38.36 | 0.84 | 1.07 |
| HSPH1 | 50.63 | 53.87 | 63.22 | 0.80 | 0.85 |
|  | |  | |  | |

**Supplementary Table 4**  Previous reports of *DNAJC7* variants in ALS

| **Reference** | **Population** | **Number of patients** | ***DNAJC7* LoF variant** | ***DNAJC7* missense variant** | **Homozygous** | **Family history** |
| --- | --- | --- | --- | --- | --- | --- |
| Farhan et al. ***Nat Neurosci*** 2019 | Mostly European | 5,095 | 8 (0.16%) | 5 (0.098%) | 0 | Not described |
| Wang et al. ***Front Genet*** 2020 | Chinese | 578 | 0 (0%) | 2 (0.35%) | 0 | No |
| Jih et al. ***Neurol Genet*** 2020 | Chinese | 325 | 1 (0.31%) | 0 (0%) | 0 | No |
| He et al. ***Neurobiol Aging*** 2021 | Chinese | 730 | 2 (0.27%) | 4 (0.55%) | 0 | No |
| Sun et al. ***Amyotroph Lateral Scler Frontotemporal Degener*** 2021 | Chinese | 326 | 0 (0%) | 2 (0.61%) | 0 | No |
| Tohnai et al. ***Neurobiol Aging*** 2022 | Japanese | 807 | 1 (0.12%) | 6 (0.74%) | 0 | No |
| Libonati et al. ***J Neurol*** 2024 | Italian | 194 | 1 (0.52%) | 0 (0%) | 0 | No information |
| Wang et al. ***Mol Neurobiol*** 2024 | Chinese | 2,124 | 1 (0.047%) | 3 (0.14%) | 0 | No |

Abbreviation: ALS, amyotrophic lateral sclerosis; LoF, loss of function

**Supplementary Data 1**

**Python code:**

from PIL import Image

import numpy as np

import pandas as pd

# Function to count the number of pixels within certain colour ranges

def count_colored_pixels(image_path):

with Image.open(image_path) as img:

img = img.convert('RGB')

data = np.array(img)

green_pixels = np.sum((data[:, :, 1] > data[:, :, 0]) & (data[:, :, 1] > data[:, :, 2]))

red_pixels = np.sum((data[:, :, 0] > data[:, :, 1]) & (data[:, :, 0] > data[:, :, 2]))

yellow_pixels = np.sum(((data[:, :, 0] > 200) & (data[:, :, 1] > 200) & (data[:, :, 2] < 100)))

total_pixels = data.shape[0] * data.shape[1]

return green_pixels, red_pixels, yellow_pixels, total_pixels

# List of image paths

image_paths = [r'C:\Users\Owner\1hikime.jpg',

r'C:\Users\Owner\2hikime.jpg',

r'C:\Users\Owner\3hikime.jpg',

r'C:\Users\Owner\4hikime.jpg',

r'C:\Users\Owner\5hikime.jpg',

r'C:\Users\Owner\6hikime.jpg',

r'C:\Users\Owner\7hikime.jpg']

# Analyse each image and store the results

results = [count_colored_pixels(path) for path in image_paths]

results_df = pd.DataFrame(results, columns=["Green Pixels," "Red Pixels," "Yellow Pixels," "Total Pixels"])

results_df['Green Ratio'] = results_df['Green Pixels'] / results_df['Total Pixels']

results_df['Red Ratio'] = results_df['Red Pixels'] / results_df['Total Pixels']

results_df['Yellow Ratio'] = results_df['Yellow Pixels'] / results_df['Total Pixels']

# Save results to an Excel file

results_df.to_excel('output.xlsx', index=False)
